# Scientific competence during medical education - insights from a cross-sectional study at a German Medical School

**DOI:** 10.1186/s12909-024-05470-7

**Published:** 2024-05-28

**Authors:** Maximilian Vogt, Nadja Schuchardt, Mark Enrik Geissler, Jean-Paul Bereuter, Rona Berit Geissler, Ingmar Glauche, Sebastian Gerdes, Andreas Deußen, Lydia Günther

**Affiliations:** 1 Division of Medical Biology, Department of Psychiatry and Psychotherapy, University Hospital Carl Gustav Carus, Technische Universität Dresden, Dresden, Germany; 2 Department of Visceral, Thoracic and Vascular Surgery, Medical Faculty and University Hospital Carl Gustav Carus, Technische Universität Dresden, Dresden, Germany; 3 Department of Physiology, Medical Faculty Carl Gustav Carus, Technische Universität Dresden, Dresden, Germany; 4 Institute for Medical Informatics and Biometry, Carl Gustav Carus Faculty of Medicine, Technische Universität Dresden, 01307 Dresden, Germany

**Keywords:** Undergraduate medical education, Scientific education, Science curriculum, Needs assessment, Research competence, Scientific skills, Curriculum development

## Abstract

**Background:**

Medical knowledge regarding the pathophysiology, diagnosis and treatment of diseases is constantly evolving. To effectively incorporate these findings into professional practice, it is crucial that scientific competencies are a central component of medical education. This study seeks to analyse the current state of scientific education and students’ desires for integration into the curriculum.

**Methods:**

From October to December 2022, a survey was distributed at the Medical Faculty Dresden to all medical students from the 1st to 5th academic year (AY). The survey investigates current expectations of applying scientific competencies later in professional life, and the students were asked to self-assess various scientific skills and in relation to the National Competence Based Catalogue of Learning Objectives for Undergraduate Medical Education. The self-assessments were objectified through a competence test with ten multiple-choice questions. The desire for curricular teaching was inquired.

**Results:**

860 students completed the survey. This corresponds to a response rate of 64%. In the 5th AY, approximately 80% of the participants stated that they expected to work with scientific literature on a daily to monthly basis in future professional life and to communicate corresponding scientific findings to patients. Only 30–40% of the 5th AY rate their scientific competencies as sufficient to do this appropriately. This corresponds with the self-assessed competencies that only slightly increased over the 5 AYs from 14.1 ± 11.7 to 21.3 ± 13.8 points (max. 52) and is also reflected in the competence test (1st AY 3.6 ± 1.75 vs. 5th AY 5.5 ± 1.68, max. 10 points). Half of the students in the 4th and 5th AYs were dissatisfied with the current teaching of scientific skills. The majority preferred the implementation of a science curriculum (56%), preferably as seminars dealing with topics such as literature research, analysis, and science communication.

**Conclusions:**

The results show discrepancies between expectations of using scientific knowledge in everyday professional life, self-rated and objectively recorded competencies, and the current state of curricular teaching of scientific competencies. There is a strong need for adequate practical training, particularly in critical analyses of scientific literature, which enables the communication of scientific knowledge to patients.

**Supplementary Information:**

The online version contains supplementary material available at 10.1186/s12909-024-05470-7

## Introduction

Scientific education and life-long learning are crucial since future generations of physicians will have to address multiple emerging medical challenges [1, 2]. It’s worth noting that, from a student perspective, scientific education is overall rather underrepresented and not mandatory in medical curricula worldwide, as highlighted by Pierre et al. [3]. Besides this scientific training is highly variable across medical schools, many programs introducing scientific education late in the curriculum and primarily in connection with doctoral theses [4, 5]. However, there are some universities around the world that have a solid integrated science curriculum in the medicine degree program [6, 7]. This results in higher attitudes towards research and more scientific productivity [8–10].

Looking more closely at the German-speaking countries in Europe, we can see that different approaches have been implemented in recent years regarding scientific training in medical curricula. In Austria, for example, a clear separation has been made since 2002 between a formal grade, which is equal for all students by the end of medical training (known as “Berufsdoktorat”), and a postgraduate and voluntary doctoral thesis or PhD [11–13]. Focusing on Germany, previous studies revealed that scientific education is not sufficiently addressed in medical curricula [14–16], especially in regular courses [17]. Scientific education mostly takes place late in the curriculum during a doctoral thesis [15]. It is important to note that a thesis project is not mandatory but is acquired by two thirds of medical students [18]. The scope and depth of scientific theses are often limited, and the MD is therefore not to be equated with the PhD [19, 20]. It is questioned whether the current system is capable of adequately preparing young physicians to make significant contributions to medical research, advance healthcare and ensure patient safety [21]. Therefore, various stakeholders, such as the German Research Foundation and the German Science and Humanities Council campaign for integrating systematic scientific training into the nationwide curriculum [22]. It has been shown that medical students themselves have a positive attitude toward science [8, 23] and wish to acquire more scientific competencies [14]. Nationwide efforts and political support are necessary for subsequent changes, and a curricular reform was initiated in 2017 [24] and may offer an opportunity to address the deficiencies in scientific education within the medical curriculum, which can differ between different medical schools. In line with this, the German Competency Based Catalogue of Learning Objectives (NKLM) [25] has been proposed as an obligatory national framework for 80% of the medical curriculum. In section VIII. 1. of this framework, scientific competencies are addressed by specific learning objectives [26]. Learning objectives and competency levels according to Miller [27] have been included. Several German medical schools have already started to implement scientific curricula and related research projects [17, 28–30], which could result in a greater number of conducted research projects as well as accepted grant applications [30]. However, such initiatives are intrinsically motivated by individual faculties and are not mandatory [31]; as far as we know, such initiatives omit the students’ perspective on curriculum development. This, however, may be an important aspect regarding needs-orientation and acceptance and has been recommended [32, 33].

At the Medical Faculty Dresden (MFD) a structured medical thesis program has been implemented since 2011 [34]. In addition, a clinician scientist program was established in 2017 [35]. While both initiatives attempt to improve scientific education, their accessibility is competitive and limited to a small number of applicants. Therefore, collaborative efforts involving educators and students are necessary to establish a science curriculum as a mandatory element for all students. We initiated a student-led investigation, supervised by several senior researchers at the MFD, to delve into the student body’s views on their actual scientific training. Recognising the crucial role that students play in shaping their educational journey, this endeavour is not only about gathering data, but also about empowering students to voice their needs and aspirations regarding their scientific education. These data can support the design and implementation of a potential standardised scientific curriculum that meets students’ expectations and needs. The primary objectives of the conducted study are as follows:

1. To assess how medical students perceive the need for scientific competencies in their future professional life and how they self-evaluate their current abilities.
2. To determine whether the students’ self-assessment of scientific competencies aligns with the currently proposed learning objectives and competence levels in the National Competence-Based for Undergraduate Medical Education (NKLM 2.0).
3. To envision what a scientific curriculum may look like based on the students’ opinions.

## Methods

### Survey design

This cross-sectional study was performed with an online survey using LimeSurvey 3 (Hamburg, Germany). The survey was designed by the authors as an iterative collaborative process and based also on other studies [14, 36, 37]. All survey items were reviewed and discussed by all group members including students from different academic years and senior scientists at the MFD. All questions were further evaluated by medical students to assess the amount of time required and to identify and remove ambiguity. The answers collected during this review process were not included in the final data analysis.

The questionnaire (Suppl. 1) consisted of eight different topics related to scientific training. We requested participant information (9 items) and asked whether the participants were interested in performing a medical thesis (4 items). We asked the students about the relevance of eight scientific competencies and how often they anticipate using them during their future professional life (ranging from daily to never). Furthermore, we asked them to self-assess their scientific skills (13 items, oriented toward the research process) according to different competence levels and in line with the NKLM: 1 (can name facts), 2 (can explain), 3a (can perform under supervision), and 3b (can perform independently). Students should also rate, if and at which level (from voluntary to mandatory) these scientific competencies should be included in the medical curriculum (17 items). Students were then asked to indicate their level of agreement on a verbal rating scale, each with specifically defined scale statements, the extent to which they agreed with the statements about satisfaction and the desire for a compulsory science curriculum. To objectify the self-assessment, a competence test was included in the questionnaire, which included ten multiple choice (MC) questions covering different science topics (science theory and method, biometry, literature, good scientific practice, clinical studies). The questionnaire ended with questions regarding the digital scientific program at MFD (2 items). The participants needed approximately 15 min on average to answer all questions.

### Distribution and data acquisition

The survey was distributed to medical students of the MFD from October to December 2022 in compulsory courses (1. AY: biology; 2. AY: physiology; 3. AY: laboratory medicine; 4. AY: general medicine; 5. AY: occupational medicine), and appropriate times were allocated for participants to complete the survey.

### Participants and data protection

The study including the experimental protocol and the terms of realisation has been approved by the Ethical committee at the Technical University of Dresden (SR-EK-152032023) as well as by the data protection officer. Only medical students of the MFD were allowed to participate in the study. Informed consent was used. The participation was voluntary. The data was collected anonymously and was stored on servers of the Technical University of Dresden. We initially obtained data from 1075 students, but only completed questionnaires were included in the subsequent analysis (*n* = 856).

### Data treatment and analysis

Python 3.8.5 was used for data analysis (Python Software Foundation, Delaware, USA) within the Visual Studio Code 1.84.0 environment (Microsoft Corporation, Washington, USA). The raw data and the source code of the performed analyses are provided in Suppl. 2 and 3.

Responses regarding previous education were categorised into healthcare-related studies (e.g., biology, psychology), healthcare-related education (e.g., physiotherapy, medical technical assistance), and non-healthcare-related education/study (e.g., economics). Parameters such as age and high school grade were analysed descriptively using means and standard deviations. Verbal rating scale questions were analysed using percentages. For the consideration of relevance in later professional work (8 items), a calculation was performed according to the following scheme: never or rarely = 0 points; monthly = 1 point; daily or weekly = 2 points. This results in a relevance sum score ranging from 0 to 16 points. For the 13 items of self-assessment, we transferred competency levels to points: can do nothing = 0 points, can name facts = 1 point, can explain = 2 points, can perform under supervision = 3 points, can perform independently = 4 points. This process results in a subjective assessment sum score ranging from 0 to 52 points. The total score was used to incorporate the students’ self-assessment into the analysis, regardless of specific items and thus to obtain a general assessment of the level of scientific education. In addition, a verbal rating scale was used to enquire about the science curriculum. We analysed the data set for relevant effects on the scientific training, the full results are presented in Supplement 4. We used the student’s t-test to assess differences between two groups. For more than two groups, we used ANOVA with post hoc Tukey’s HSD. The level of significance was set to alpha = 0.05.

In our analysis, box plots depict the interquartile range, with whiskers extending to 1.5 times the interquartile range and the median distinctly marked inside the box.

## Results

### Sample characteristics

Out of 1333 enrolled medical students a total number of 1075 (80.6%) participated in the survey. A total of 856 complete responses were included in further analyses (Table 1). In four of the five AYs, we achieved response rates > 50%. Overall, nearly half of the students (48.4%) had already completed health-related training before starting medical school, this differs strongly between academic years.

**Table 1:** Survey sample distribution and demographic data per academic year

	1st academic year	2nd academic year	3rd academic year	4th academic year	5th academic year
Enrolled students	229	223	293	294	294
Participating Students, n	215	87	219	160	175
Response rate (%)	93,9	39	74,7	54,4	59,5
Sex (n, %)					
Male	65 (30.2%)	17 (19.5%)	73 (33.3%)	48 (30%)	58 (33.1%)
Female	149 (68.3%)	70 (80.5%)	145 (66.2%)	111 (69.4%)	116 (66.3%)
Diverse	1 (0.5%)	0	1 (0.5%)	1 (0.6%)	1 (0.6%)
Age, in years (Mean, SD)	21.8 ± 2.7	23.2 ± 3.7	23.1 ± 2.7	24.3 ± 3.2	25.2 ± 2.9
High school GPA (Mean, SD)	1.56 ± 0.52	1.56 ± 0.48	1.54 ± 0.42	1.50 ± 0.48	1.51 ± 0.40
Promotion (n, %)					
Strives for it	140 (65.1%)	67 (77.0%)	173 (79.0%)	57 (35.6%)	31 (17.7%)
Not planned	3 (1.4%)	2 (2.3%)	5 (2.3%)	9 (5.6%)	10 (5.7%)
Started	1 (0.5%)	1 (1.1%)	6 (2.7%)	70 (43.8%)	113 (64.6%)
Don´t know	69 (32.1%)	17 (19.5%)	35 (16.0)	20 (12.5%)	18 (10.3)
Cancelled	0	0	0	2 (1.3%)	1 (0.6%)
Others	2 (0.9%)	0	0	1 (0.6%)	2 (1.1%)
Educational background (n, %)					
None	81 (37.7%)	25 (28.7%)	122 (55.7%)	96 (60.6%)	116 (66.3%)
Healthcare-related education	118 (54.9%)	59 (67.8%)	81 (37.0%)	45 (28.1%)	42 (24.0%)
Healthcare-related study	11 (5.1%)	3 (3.5%)	10 (4.6%)	15 (9.4%)	13 (7.4%)
Non-healthcare education and study	5 (2.3%)	0	6 (2.7%)	4 (2.5%)	4 (2.3%)

### Scientific skills—assessment of relevance

**Fig. 1:**
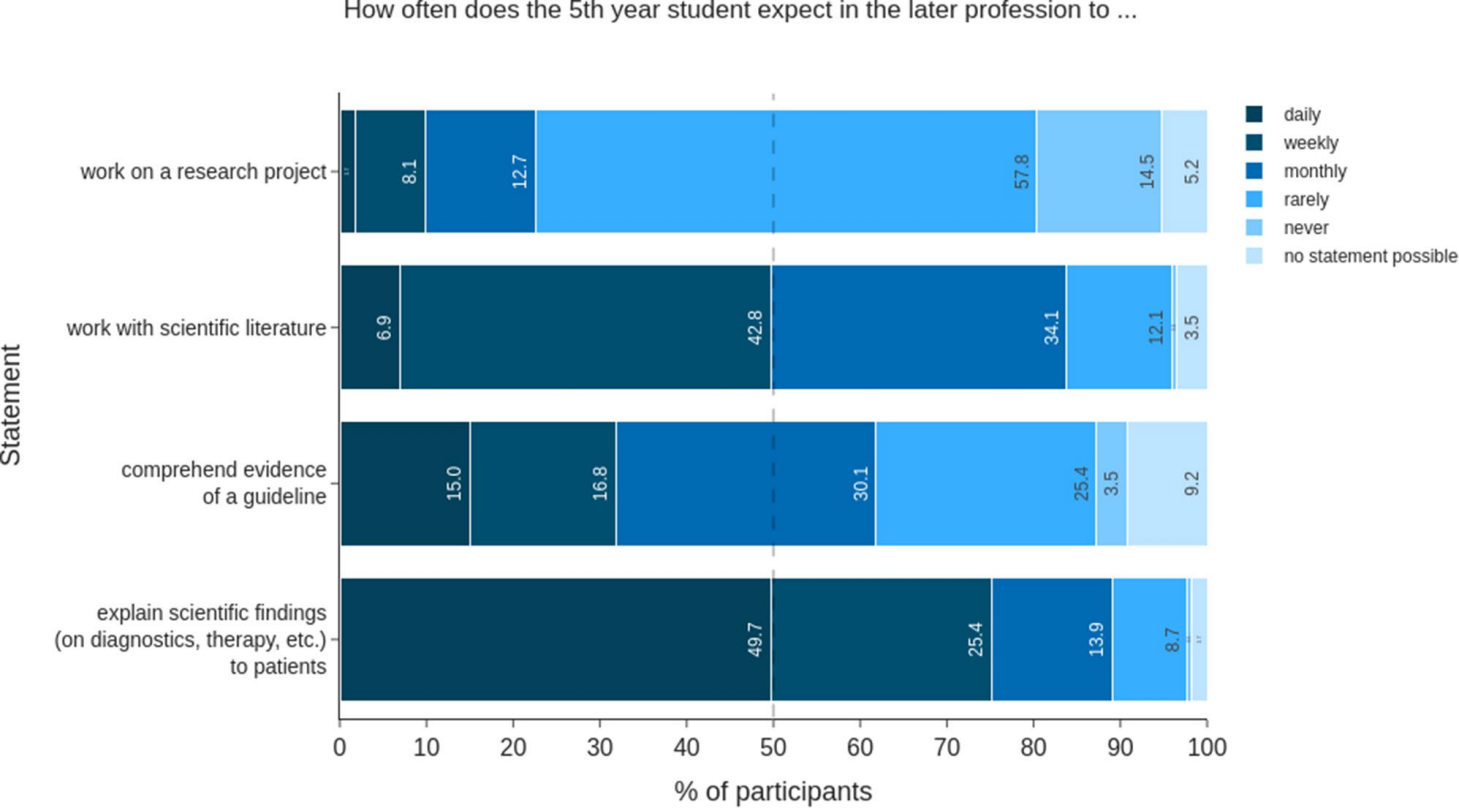
Assessment of the need for scientific skills among 5th AY students. Students (*n* = 175) were asked about specific skills regarding their expected frequency in later professional life. The responses are presented as a stacked bar chart in percentages. The 50% mark is denoted by a dashed line

To assess the subjective importance of different scientific skills in future daily work, responses from 5th AY students (*n* = 175) are presented (Fig. 1). Some scientific skills were considered to be more relevant than others: working on research projects was expected to be a less frequently needed skill. It was chosen by approximately 21% of respondents on a monthly or more frequent basis, compared to over 70% who expected to use it less often. Nearly half of the students (47.7%) expected to work with scientific literature either daily or weekly in their medical practice; 34.1% expected to do so at least monthly. Comprehending the evidence of a guideline was perceived as relevant by approximately two-thirds (61.9%) and as an activity that would later be performed monthly or more frequently. Explaining scientific evidence about diagnosis and treatment to patients was considered relevant at least monthly by 89% of the students.

### Scientific education—satisfaction across academic years

**Fig. 2:**
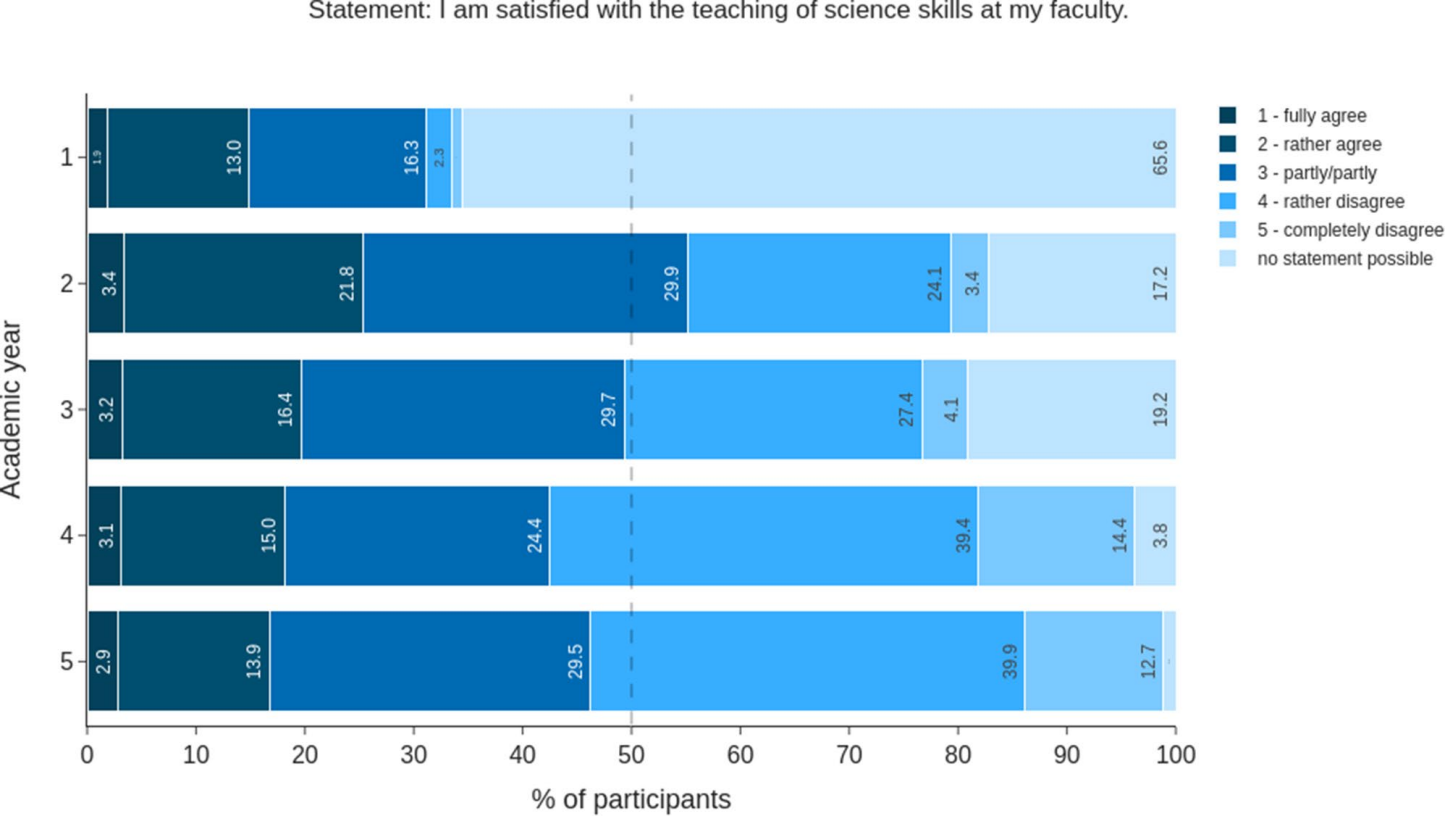
Assessment of satisfaction with the current scientific curriculum across AYs. The responses of all the students are shown (*n* = 856). The responses are presented as a stacked bar chart in percentages. The 50% mark is denoted by a dashed line

**Fig. 3:**
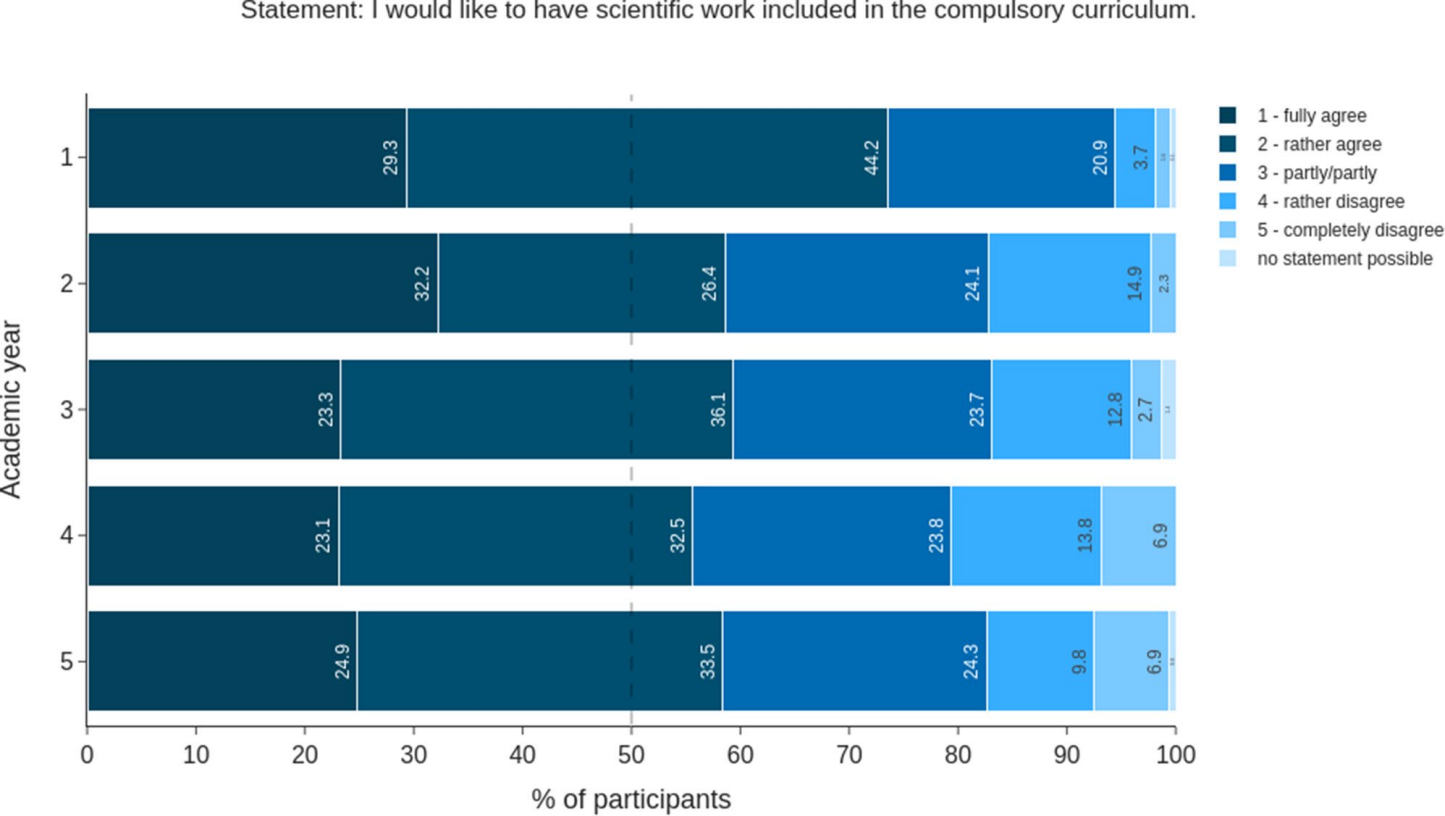
Evaluation of the introduction of a compulsory curriculum for scientific education. The answers of all students are shown (*n* = 856). The answers are shown as a stacked bar chart in percentages. The 50% mark is denoted by a dashed line

The satisfaction with the teaching of scientific skills differed among AYs. In the first AY, two-thirds of the students (65.6%) chose “no statement possible”. This option was chosen by 20% or less in AY 4 and AY 5. While every fourth student (27.5%) in the second year of study responded ‘rather disagree’ or ‘disagree’ to the statement ‘I am satisfied with the teaching of scientific skills at my faculty’, more than half (52.6%) of the 5th AY students did so (Fig. 2). The percentage of students who were completely satisfied remained constant at approximately 3% from the second year onwards. In addition, most students (> 50%) in each AY agreed fully or partly with the statement “I would like to have scientific work included in the compulsory curriculum” (Fig. 3).

 Medical thesis - influencing factors for intensive scientific training - this chapter has been removed from the originally uploaded manuscript.

### Scientific skills – self-assessment compared to different competence levels

**Fig. 4:**
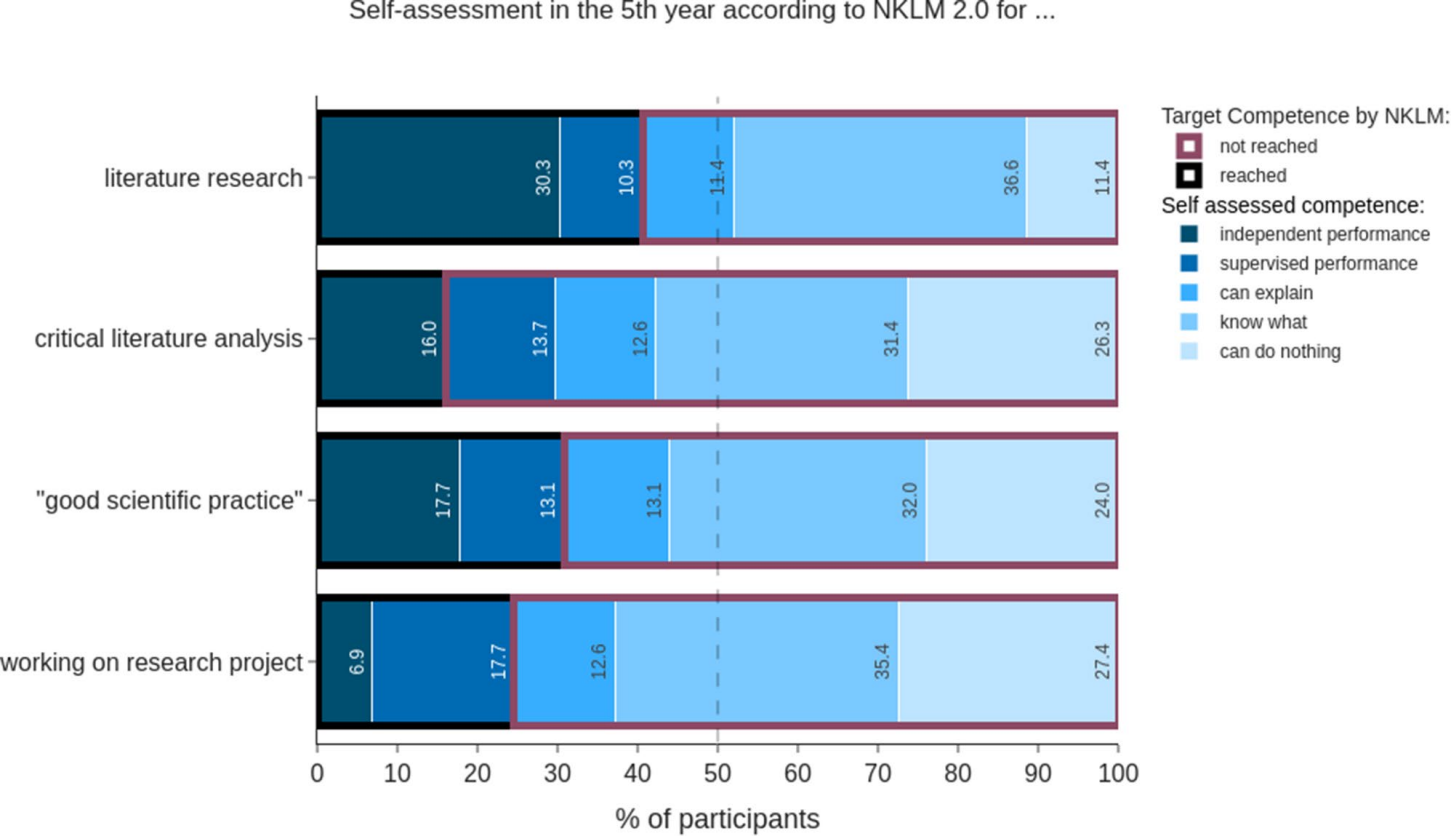
Self-assessed competencies for different scientific skills in the 5th AY. The self-assessment is plotted horizontally. The percentage of students who achieved the competence target required is shown by a black outline versus not in red

We compared the self-assessments of 5th year students (*n* = 175) to different competence levels and to a proposed catalogue of learning objectives for the national medical curriculum. Working with scientific literature was rated by more than 80% of all students as a relevant, at least monthly, skill used in later professional life (Fig. 1). Despite the high relevance, 59.4% of the 5th AY students rated themselves clearly below the level of being able to perform under supervision or independently and therefore below the targeted NKLM level (Fig. 4). This competence is also highly relevant to doctoral students. Among all 5th-year students, > 50% rated themselves as not being able to work under supervision or independently regarding good scientific practice and working on a research project (Fig. 4). Overall, a substantial number of students (between ∼ 10–30%, depending on the competence addressed) state “0” in the 5th AY, which means they assess themselves as “can do nothing”.

### Identification of factors that influence differences in scientific attitude

**Fig. 5:**
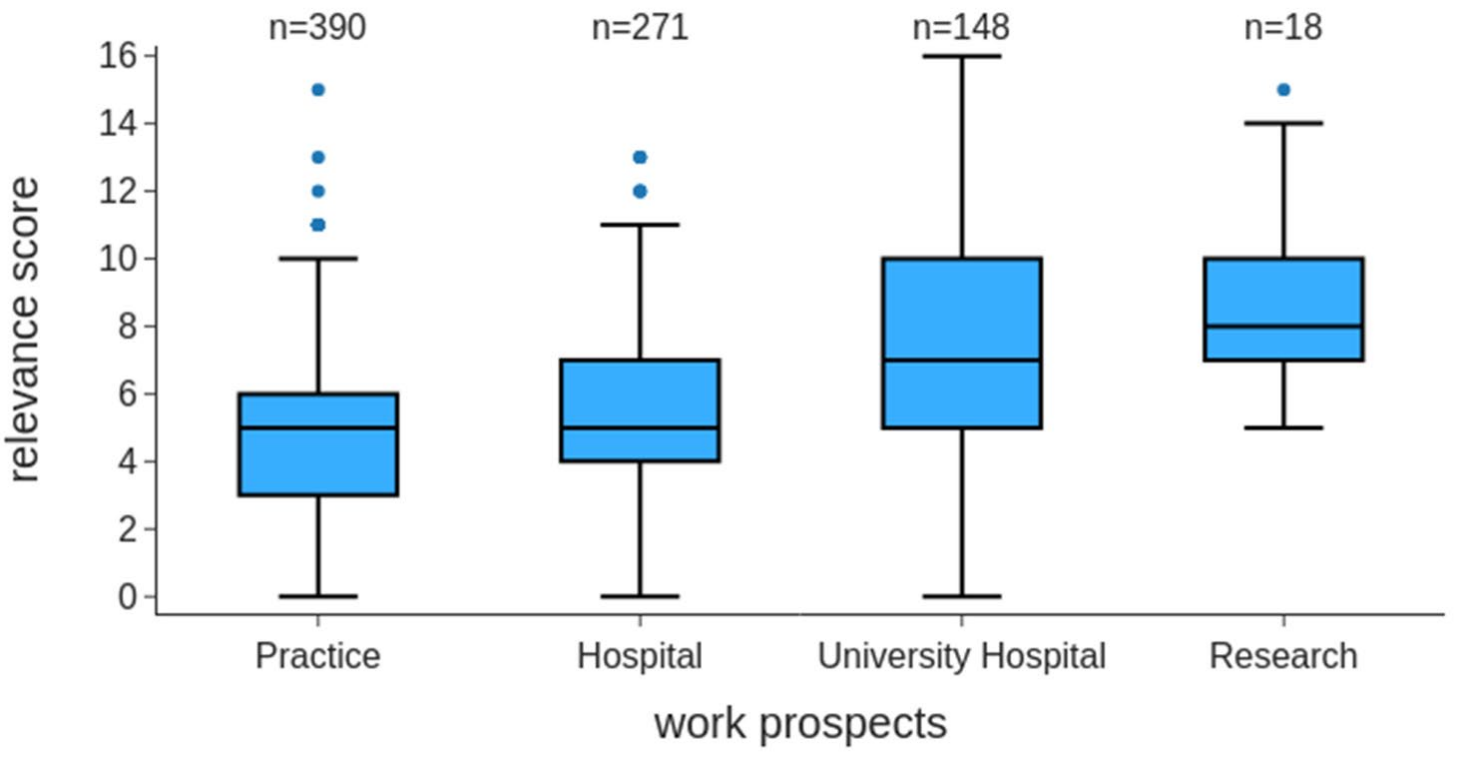
Box plots for the relevance score of scientific competences (0–16) on later career aspirations. Assessments of the relevance of scientific competences for later careers: practice (5.0 ± 2.5, *n* = 390), clinic (5.4 ± 2.5, *n* = 271), university hospital (7.6 ± 3.4, *n* = 148), and research (8.7 ± 2.7, *n* = 17). Significant differences among these groups were found, with specific differences identified between certain categories

We found relevant differences for the factor “later career aspirations” in the context of the cumulative relevance score. We discovered that among all participants (*n* = 856), those who saw themselves either in research (*n* = 18) or in a university hospital (*n* = 148) tended to rate scientific training as more relevant than those with a preference for practice (*n* = 390) or hospital (*n* = 271). An ANOVA revealed a significant difference in relevance scores across the four groups (F(3, 823) = 43.11, *p* < 0.001). The subsequent Tukey’s HSD post hoc test showed significant differences between hospital and research (difference between the means = 3.35, *p* < 0.001), hospital and university hospital (difference between the means = 2.23, *p* < 0.001), practice and research (difference between the means = 3.76, *p* < 0.001), and practice and university hospital (difference between the means = 2.63, *p* < 0.001). However, a clear majority (77.2%) of the participants saw themselves either in practice or in a nonuniversity hospital and rated scientific training as less relevant for later professional life (Fig. 5).

**Fig. 6:**
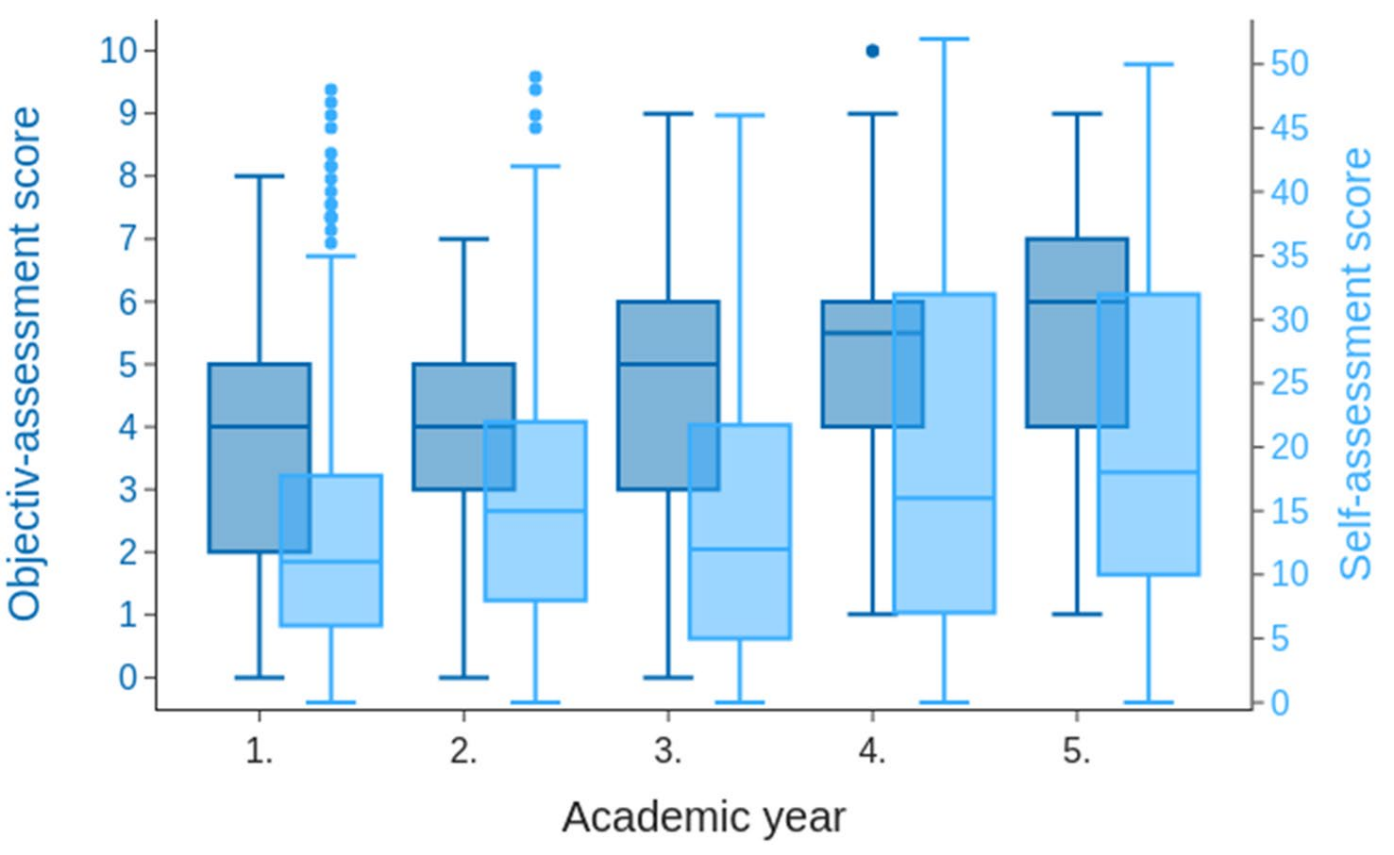
Box plots for objective and self-assessment score distributions. The box plots show both the objective assessment (0–10) in dark blue on the left y-axis and the subjective assessment (0–52) in light blue on the right y-axis. The distribution of scores is plotted over the academic years

**Fig. 7:**
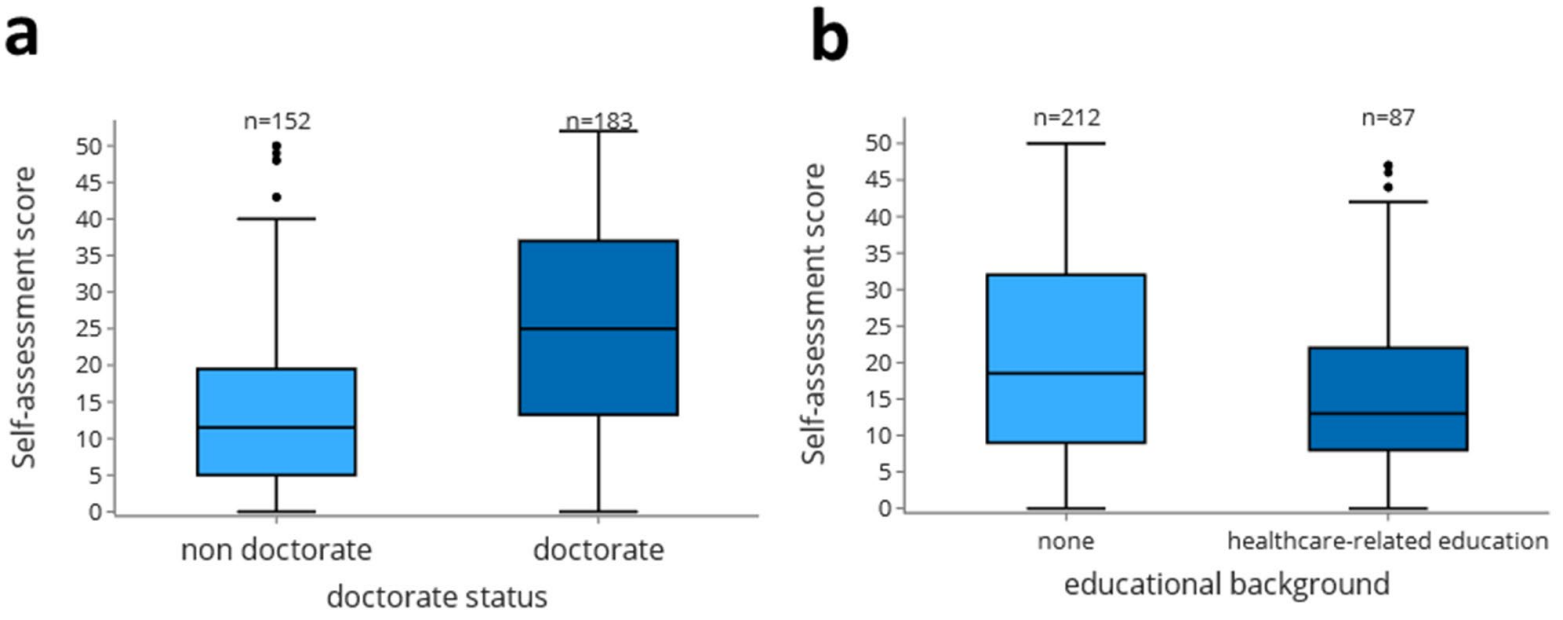
Factors that influence the self-assessment score. (**a**) Box plots for self-assessed competences (0–52 points) by doctoral status in the 4th and 5th AY (Non-doctoral students vs. current doctoral students) (**b**) Box plots for self-assessment of competences by educational background in the 4th and 5th AY of study. Students with no previous education and healthcare-related education were compared

To measure the subjective level of current scientific knowledge, a 13-item self-assessment score was collected (Fig. 6). The self-assessment showed a high variance in all AYs (minimum SD = 11.1), indicating the heterogeneity of the students’ scientific knowledge while also having a high Cronbach’s alpha (Cronbach’s alpha = 0.947, 95% CI = 0.942–0.952). However, an increase of 7.0 points (max. 52 points) was observed from the 1st to the 5th AY (coef.=0.87/academic semester, CI = 0.59–1.16). By modelling the factors that led to a higher degree of self-assessment in a general linear model (details about the fitted model are provided in the supplementary material), we were able to identify two strongly associated factors: pursuing a doctoral degree was associated with a higher self-assessment (median non doctorate 11.5 [interquartile range 5.0-19.3] vs. median doctorate 25.0 [interquartile range 13.5–37.0], *p* = 0.000; Fig. 7a), and on the contrary, having previous medical education was associated with a lower self-assessment (median no previous education 18.5 [interquartile range 9.0–32.0] vs. median healthcare-related education 13.0 [interquartile range 8.0–22.0], *p* = 0.006; Fig. 7b). The objective knowledge test included ten questions (Cronbach’s alpha = 0.374, 95% CI = 0.310–0.435). Between the 1st and the 5th AY, an improvement of 2.0 points from a maximum of 10 points was achieved. Over the AYs, there was a marked increase in the number of correct answers to questions on bias, scale types and, to a lesser extent, level of evidence, particularly after students had attended the Biometry class, where these topics were covered (Suppl. 5).

### Perspective of curricular development—students’ wishes

**Fig. 8:**
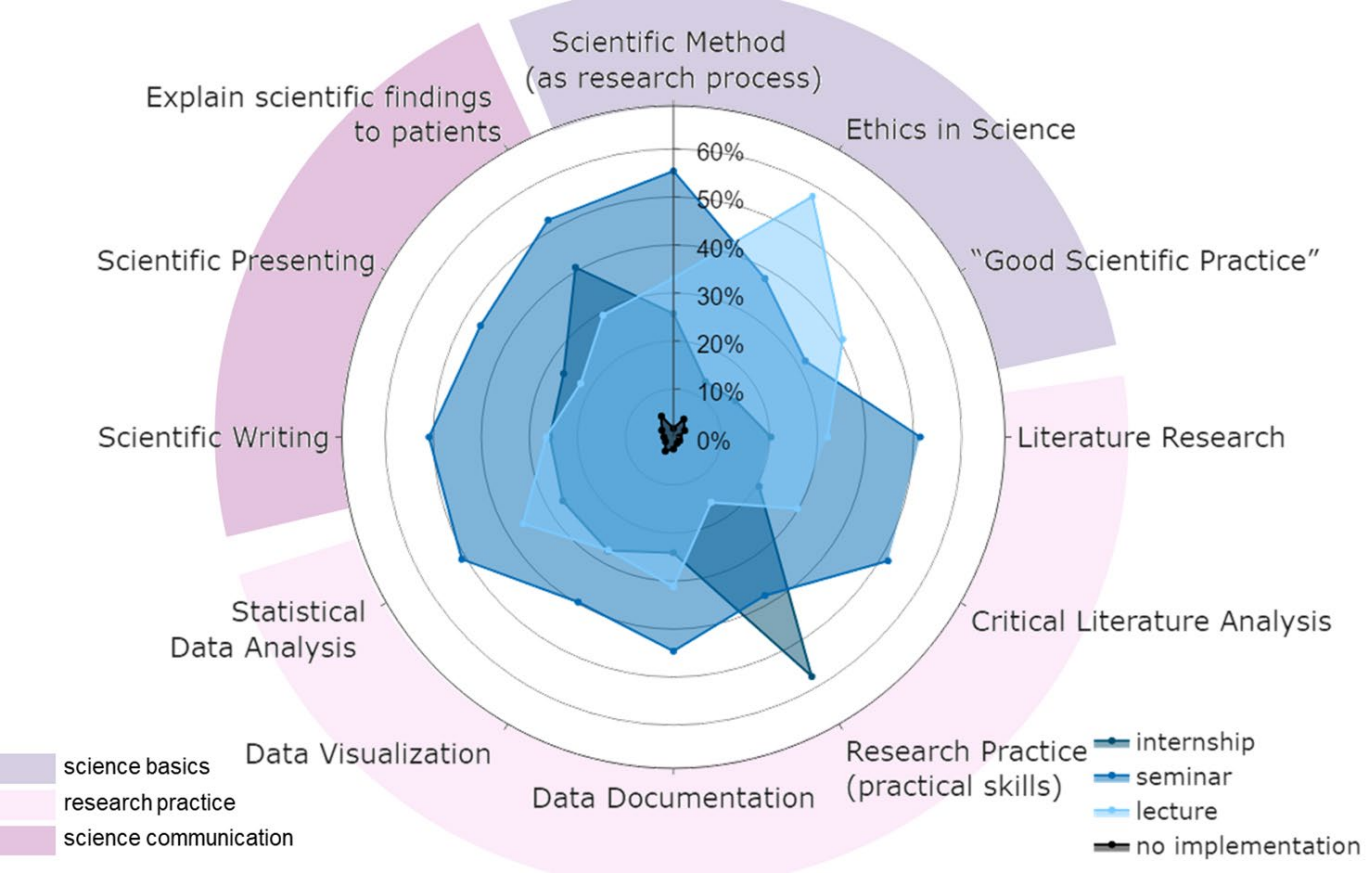
Students’ wishes for a scientific curriculum. The polar diagram shows the rankings of 12 items for a planned science curriculum as rated by students (multiple choices possible) for implementation in the curriculum (bottom right legend). The items were grouped into 3 content blocks (bottom left legend)

Finally, the students were asked to indicate their preferred form of teaching for a range of topics in science education (Fig. 8). Students were allowed to make multiple choices (lecture, mandatory seminar or internship, and elective). We could see indications of more scientific content in a student-based curriculum: for most of the given content, including “scientific method”, “literature research”, “statistical data analysis”, and “scientific writing”, the mandatory seminar was the preferred teaching format (∼ 50%). The majority preferred a lecture format on ethics in science (57.9%), while practical training was aspired to learn practical research skills (57.6%) Fig. 8.

## Discussion

We present the results of a systematic assessment of the students’ perspectives on scientific education in medical training at the MFD, obtained in 2022. This has been done in preparation for the upcoming intended implementation of mandatory scientific training in the medical curriculum. This allows us to draw conclusions on how curricular change processes could be organised according to the needs of the target group. In the long term, continuous monitoring could contribute to evidence-based curricular development, which means: “Is the intervention successful and do the students really improve in scientific competencies?”. We highly recommend a follow-up in which various outcomes are measured again with a questionnaire, including self-assessment as well as other endpoints like quantity and quality of thesis projects. Furthermore, we strongly encourage those involved in curricular development or educational research to involve students in this process. This is valuable for the curriculum to be planned and enables students to engage in all steps of research-based learning.

### Expectations versus reality

The results of our survey showed substantial discrepancies with regard to the expected necessity of scientific competence in later professional life and the self-assessment of skills in the scientific field. In this analysis we focused on 5th AY because they are about to graduate and enter the medical profession as well as they should have acquired scientific competence by then. The majority of 5th AY students estimated that they would need scientific skills, such as working with and critically reflecting scientific literature, very frequently. Most students expected that their medical studies would enable them to interpret scientific results but evaluate their training as insufficient (Figs. 1 and 4), which has also been shown elsewhere [38]. In a nationwide survey, approximately 93% agreed or rather agreed that the critical analysis of scientific publication is a key competency for physicians [14]. However, most of our students rated themselves as low performers and clearly below the required level of being able to work independently. We also analysed how the 5th AY students regarded their self-assessment of good scientific practice and working capabilities in a research project. Again, we found that only one in four students considered themselves sufficiently educated. This is evidence of an insufficient competence level in graduates with respect to independent work in a doctoral thesis. Our results are in line with previous studies showing insufficient self-assessed scientific competencies in medical students, especially in terms of scientific methods and writing, practical training and statistics [14, 15].

Students younger than the 5th AY answered quite similarly (data not shown), thus supporting the results presented. Since there was no substantial change in the curriculum at our faculty over recent years, one can expect that the students who graduate in the next few years will also not be sufficiently trained in scientific skills. Thus, from the student’s perspective, there is an urgent need to establish mandatory scientific training in the curriculum. There are different ways to implement this approach, but research-based learning [39–41] is one possibility for integrating scientific thinking in existing curricula. In line with this, Eckel et al. (2019) showed that slight modifications of a practical training course in physiology could improve scientific thinking in medical students without disturbing knowledge acquisition [28].

### Impact of doctoral thesis and previous education

We observed that students who were performing a doctoral thesis rated their skills overall better than did those who were not involved in a medical thesis **(**Fig. 7a**)**. This indicates that actively working on a scientific project could positively affect the subjectively perceived increase in knowledge regarding scientific topics, as shown previously [18]. In detail, among students involved in a thesis project, self-assessment with respect to methodology and practical methods increased, but scientific writing seemed difficult, even while performing a medical thesis [15]. Overall, approximately two thirds of all medical students complete a doctoral thesis in Germany [18, 42] and approximately 210 students graduate per year at our faculty (personal communication, Prodekanat Forschung). Many medical doctoral theses are driven primarily by extrinsic factors [36, 38] and the lack of intrinsic motivation may be due to the lack of scientific training program and supervision [14, 43–45]. In this regard, Kuhnigk et al. [4] and Pfeiffer et al. [46] showed that graduate programs and professional training have an impact on the success and quality of medical theses. However, only ∼ 15% of doctorate students are enrolled in graduate programs, although approximately 25% would like to attend such courses [38]. Similarly, the MFD offers a structured graduate program to medical students [34], but participation is based on competition for admission and reserved for a small number of students (∼ 15 per year). Therefore, mandatory scientific theoretical and practical training for all students could improve the overall quality of medical theses. In addition, the introduction of a science-oriented longitudinal curriculum that teaches basic scientific concepts from the first semester onwards should be seen as part of the fundamental basis of medical training [47]. On the one hand, this can promote the scientific outcome of the postgraduates [48, 49] with regard to research output and may also increase patient safety and professional care [10, 50]. However, there is currently a lack of long-term evidence that the teaching of evidence-based medicine has a strong influence on subsequent student behaviour [51].

The students who had a previous medical education background before starting medical studies rated themselves less favourable according to the subjective assessment (Fig. 7b). This could indicate that medically trained people already have an impression of the complexity of certain scientific topics. Older age and previous educational experience are associated with increased accuracy in self-assessment [52]. Our data showed that this primarily affects self-assessment, as we could not observe the same effects on objective competency test scores (data not shown).

### Self-assessment versus competency test

The overall subjective assessment showed a high variance in the different AYs. We observed a slight increase in self-assessed abilities across AYs (Fig. 6); however, even in the higher AYs, the third quartile was only 32 points out of a maximum of 52 points (61.45%). This indicates a high level of uncertainty even among the more experienced students. It should be acknowledged that self-assessments are discussed critically in the literature and should not be used as the only criterion for assessing learning outcomes. Especially at the beginning of studies, such self-assessment may not be valid [53]. For this reason, we focused on the answers of higher AYs to certain questions. We also observed that the majority of the 1st AY students who were at the beginning of their studies answered the question about satisfaction with the current science curriculum with “no statement possible”. This shows that some of the students had a realistic assessment of the statements they were able to evaluate objectively, and this is useful in the context of internal control. Approximately 20% of the students in the 2nd and 3rd AYs also chose this option, yet < 5% of those in the 4th and 5th AYs chose this option. Nevertheless, the data from the early AYs seem useful for longitudinal follow-up, making it worth including them in such analyses.

To substantiate the self-assessment, we conducted a ten-question competence test covering various scientific topics (see Suppl. for details). Here, we observed an increase in the average score (2.4 points; Fig. 6**)** from approximately 4 points (1st AY) to approximately 6 points (5th AY). In parts, we observed large differences in the correct answers to individual questions. There was a large amount of knowledge deficit with respect to the inductive research approach (< 20% correct answers), the scientific method (as the research process) and good scientific practice (∼ 35–40% correct answers). These deficits remained constant over AYs (data not shown). Obviously, these topics were not addressed sufficiently during the course of the curriculum. This finding fits well with the self-assessment of the 5th AY, where only approximately 25–30% of the participants rated themselves as sufficiently trained to perform research either independently or at least in a supervised environment. In contrast, more than 50% could answer the question about the components of an abstract correctly from the first AY onwards, increasing to almost 80% in the fifth AY, indicating that this content had been successfully implemented. Questions addressing aspects of clinical research, i.e., randomisation, bias and scale types, displayed a clear trend over the AYs. With the 3rd AY onwards, there was a substantial increase in knowledge, which is in line with already implemented courses addressing biometry and statistics.

However, the limited number of questions and the low Cronbach’s alpha indicate that the test results can provide information only about topics that might be addressed more comprehensively in the curriculum. Notably, there were no differences between the sexes according to either the self-assessment or the objective competence test. This finding aligns with the marginal sex effects observed in the Bavarian Graduate Study in Medicine [54].

### Students’ wishes and best practices

Looking more closely at the statement “I am satisfied with the teaching of scientific knowledge at my faculty”, it becomes clear that satisfaction decreases with the experience of studying **(**Fig. 2**)**. Finally, approximately 50% of the 5th AY quoted that they were satisfied or rather satisfied. This is even more alarming, as approximately 65% of our 5th AY cohort had already started a doctoral thesis, and a further approximately 18% would like to do so (data not shown). In a previous study Ratte et al. [14] showed that 79% of the students surveyed stated that they were not or rather not well prepared for working on a dissertation during their studies and that they wished for more training covering different scientific topics. This raises the question of how to design a future curriculum that improves student satisfaction, self-assessment, and in particular objective competence. Besides “Ethics in science” and “Good scientific practice”, the compulsory seminar was chosen as the favoured teaching format by our students for all the other topics (Fig. 8). This is interesting since at the MFD, the curriculum is already tightly timed, and seminars serve to discuss particularly important topics relevant to the majority of students. This finding may confirm the importance attributed to scientific literacy by the students, as the target group knows its own curriculum best. The 5th AY is a valid group for such statements from a retrospective point of view, and we recommend the regular involvement of this experienced group during curriculum development. However, the didactic expertise of even higher AY’s students must be questioned at this point. Scientific work is learned in practice, and experts recommend that scientific teaching should take place in a “real” environment - supplemented by journal clubs or other teaching formats [47, 48]. For this reason, the Federal Ministry of Health’s draft bill for the new licence to practise medicine published at the end of 2020 also provides for a mandatory 12-week period of practical research work which is to be implemented [55]. Lectures and occasional internships were also chosen (multiple answers possible), but they were chosen considerably less often. The option to “not anchor” a specific topic in the curriculum received less than 10% of the votes for any of the subject areas. Overall, our students wished for interactive student-centred formats, making discussions possible and leading to a well-grounded scientific education, which was also shown in other studies [14]. The addition of scientific training to an already densely packed curriculum without sacrificing other content is questionable and widely debated [47, 56]. We emphasise the importance of intrinsic and extrinsic motivation [57] and voluntary engagement, highlighting that this is a matter of attitude rather than simply increasing workload. It’s crucial to teach and inculcate this mindset differently in terms of a professional attitude. In addition, students should be asked about their willingness to invest time and effort into this subject at the expense of other subjects.

### Outlook and next steps

Due to the existing curriculum and limited possibilities for including new curricular content, we propose several steps to establish a longitudinal scientific curriculum on the basis of a needs assessment: (1) structured analysis of the curriculum and mapping to learning objective frameworks such as graduate profiles or the NKLM, (2) integration of the students’ expectations into the curriculum, and (3) implementation accompanied by content training of faculty members regarding the basics of scientific working. This should be accompanied by assessing structural and/or financial needs to ensure that curriculum goals and resources are congruent with each other. Furthermore, we strongly suggest implementing digital courses, which could accompany development. The students’ wish for interactive formats could be met without overloading the curriculum since familiarisation with the content could be performed individually (which will require saved time) followed by student-centred discussions tutored by experienced faculty members. In general, students need to be highly intrinsically motivated to learn the basics of science education, and so far, there has been little research into what student engagement might look like in concrete terms, e.g., time.

### Limitations

Our study faced several limitations. First, a new selection process has recently been implemented and with the new selection process the fraction of students with a previous medical or non-medical educational background increased in the 1st and 2nd AY in comparison to the 3rd to 5th AYs, which were selected differently. Also in the 3rd AY, about 80 students per year come from other universities to continue their studies at the MFD. Thus, the homogeneity between the AYs can therefore be questioned. This is reflected firstly in the number of enrolled students (Table 1) and secondly in terms of previous curricular experience, which may have influenced the self-assessed ratings as well as the competence test. This limitation could not be avoided in the current assessment. Importantly, the current study can serve as a starting point for annual or at least repetitive investigations in which the annual cohort serves as its own control. As such, the potential effects of a longitudinal heterogeneity would cancel out. While our approach of surveying during mandatory courses ensured a commendable response rate, there was a noticeable decrease in participation during the third semester (second AY) due to illness of the course coordinator. Nevertheless, the response rate was close to 40%, which appears reasonable for a representative poll. Additionally, the relationship between self-assessment and objective competencies is tenuous at best, with some studies suggesting little to no correlation [53], The competency test applied in our study was time-constrained, limiting its robustness and, consequently, its inferential power. We used diagnostic graphs to convert the self-assessment scores to a linear scale. While this mathematical transformation aids analysis, it opens up a discussion about whether such linearity can be applied appropriately to the inherently multifaceted nature of skills and competences. Finally, we did not ask the students how much time they would dedicate to their own scientific skills development. Due to the aim of independent scientific work and thinking, there is a significant need for practical skill acquisition, which is mostly achieved through hands-on experience. Although the limitations described above must be taken into account when interpreting the results, our database is a rich resource and a valid means of addressing a range of needs in the implementation of a science curriculum.

## Conclusion

Our study underscores the importance of rigorous scientific education in medical education. There is a clear request by students for the acquisition of necessary scientific competencies based on a mandatory science curriculum. In preparation for this, we highly recommend a needs assessment by means of a survey to establish a longitudinal research curriculum and to ensure that the implementation of the curriculum is successful and well accepted by students and teaching staff. Furthermore, this should be done on a nation-wide basis.

### Electronic supplementary material

Below is the link to the electronic supplementary material.

Supplementary Material 1

Supplementary Material 2

Supplementary Material 3

Supplementary Material 4

Supplementary Material 5

Supplementary Material 6

## Data Availability

Data is provided within the manuscript or supplementary information files.

## Abbreviations

AY: academic year
Fig.: Figure
MFD: medical faculty Dresden
NKLM: German Competency Based Catalogue of Learning Objectives
Suppl.: Supplement
