# Supplementary figures and images for: Scientific competence during medical education - insights from a cross-sectional study at a German Medical School
Source: BMC Med Educ. 2024 May 28;24:590. doi: 10.1186/s12909-024-05470-7 (PMC11134709; doi:10.1186/s12909-024-05470-7)

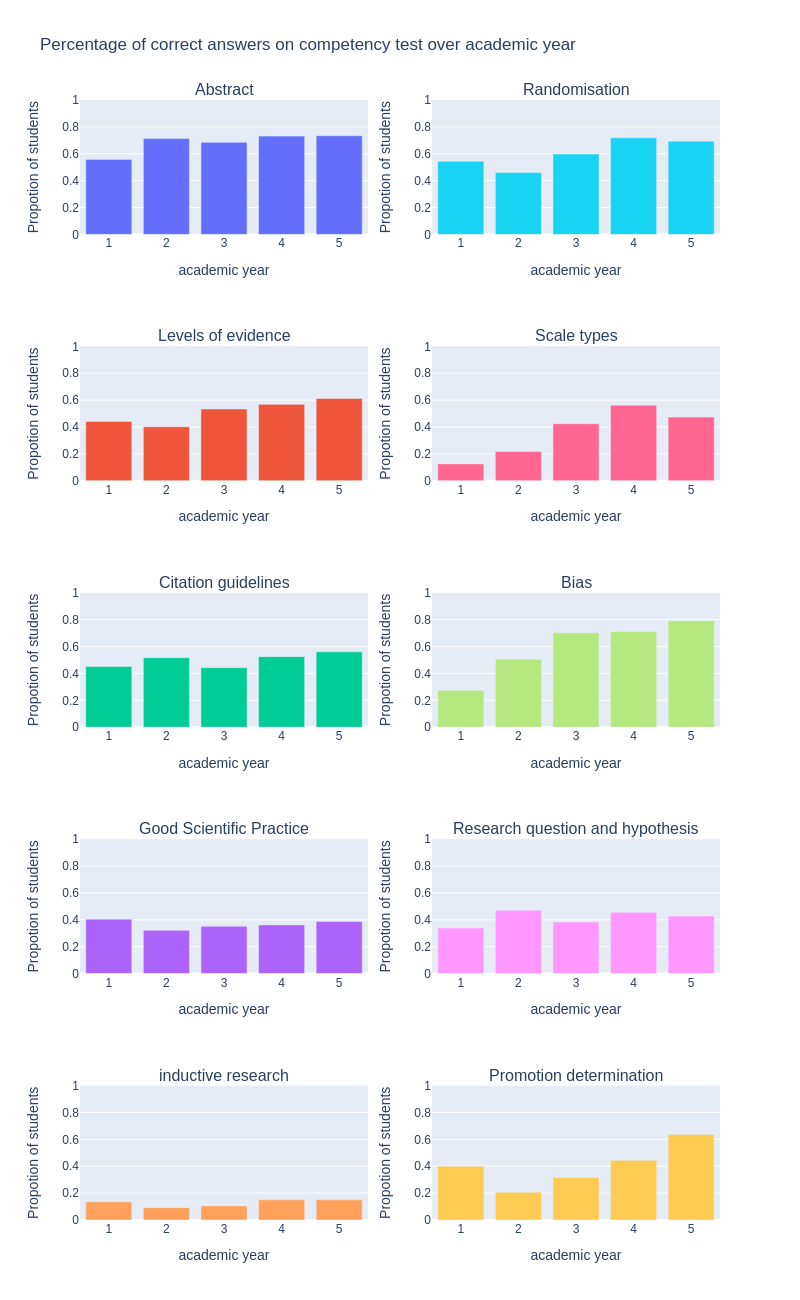

Supplement: Supplementary file 4 — Supplementary Material 4 [file 12909_2024_5470_MOESM4_ESM.png]
