# Supplementary material for: Scientific competence during medical education - insights from a cross-sectional study at a German Medical School
Source: BMC Med Educ. 2024 May 28;24:590. doi: 10.1186/s12909-024-05470-7 (PMC11134709; doi:10.1186/s12909-024-05470-7)

| All entries = 980 | First Year of Study | Second Year of Study | Third Year of Study | Fourth Year of Study | FIfth Year of Study |
|---|---|---|---|---|---|
| Enrolled Students | 229 | 223 | 293 | 294 | 294 |
| Participating Students, n | 230 | 103 | 260 | 217 | 218 |
| Response Rate (%) | 100 | 46.2 | 88.7 | 73.8 | 74.1 |
| Sex (n, %) | | | | | |
| Male | 68 (30.9%) | 21 (20.3%) | 85 (32.7%) | 60 (27.6%) | 71 (32.5%) |
| Female | 160 (69.6%) | 80 (77.6%) | 173 (66.5%) | 155 (71.4%) | 144 (66.0%) |
| Diverse | 1 (0.4%) | 1 (0.9%) | 1 (0.4%) | 2 (0.9%) | 2 (0.4%) |
| Age, in years (Mean, SD) | 21.8 ± 3.2 | 23.0 ± 3.6 | 23.3 ± 3.0 | 24.4 ± 4.3 | 25.0 ± 2.9 |

| only complete answers =856 | 1st academic year | 2nd academic year | 3rd academic year | 4th academic year | 5th academic year |
|---|---|---|---|---|---|
| Enrolled Students | 229 | 223 | 293 | 294 | 294 |
| Participating Students, n | 215 | 87 | 219 | 160 | 175 |
| Response Rate (%) | 93.9 | 39.0 | 74.7 | 54.4 | 59.5 |
| Sex (n, %) | | | | | |
| Male | 65 (30.2%) | 17 (19.5%) | 73 (33.3%) | 48 (30%) | 58 (33.1%) |
| Female | 149 (68.3%) | 70 (80.5%) | 145 (66.2%) | 111 (69.4%) | 116 (66.3%) |
| Diverse | 1 (0.5%) | 0 | 1 (0.5%) | 1 (0.6%) | 1 (0.6%) |
| Age, in years (Mean, SD) | 21.8 ± 2.7 | 23.2 ± 3.7 | 23.1 ± 2.7 | 24.3 ± 3.2 | 25.2 ± 2.9 |
| High School GPA (Mean, SD) | 1.56 ± 0.52 | 1.56 ± 0.48 | 1.54 ± 0.42 | 1.50 ± 0.48 | 1.51 ± 0.40 |
| Promotion (n, %) | | | | | |
| strives for it | 140 (65.1%) | 67 (77.0%) | 173 (79.0%) | 57 (35.6%) | 31 (17.7%) |
| not planned | 3 (1.4%) | 2 (2.3%) | 5 (2.3%) | 9 (5.6%) | 10 (5.7%) |
| started | 1 (0.5%) | 1 (1.1%) | 6 (2.7%) | 70 (43.8%) | 113 (64.6%) |
| dont know | 69 (32.1%) | 17 (19.5%) | 35 (16.0) | 20 (12.5%) | 18 (10.3) |
| cancelled | 0 | 0 | 0 | 2 (1.3%) | 1 (0.6%) |
| others | 2 (0.9%) | 0 | 0 | 1 (0.6%) | 2 (1.1%) |
| Educational Background (n,%) | | | | | |
| none | 81 (37.7%) | 25 (28.7%) | 122 (55.7%) | 96 (60.6%) | 116 (66.3%) |
| Healthcare-related Education | 118 (54.9%) | 59 (67.8%) | 81 (37.0%) | 45 (28.1%) | 42 (24.0%) |
| Healthcare-related Study | 11 (5.1%) | 3 (3.5%) | 10 (4.6%) | 15 (9.4%) | 13 (7.4%) |
| Non-healthcare Education and Study | 5 (2.3%) | 0 | 6 (2.7%) | 4 (2.5%) | 4 (2.3%) |

with 980 (all those who have answered this question)

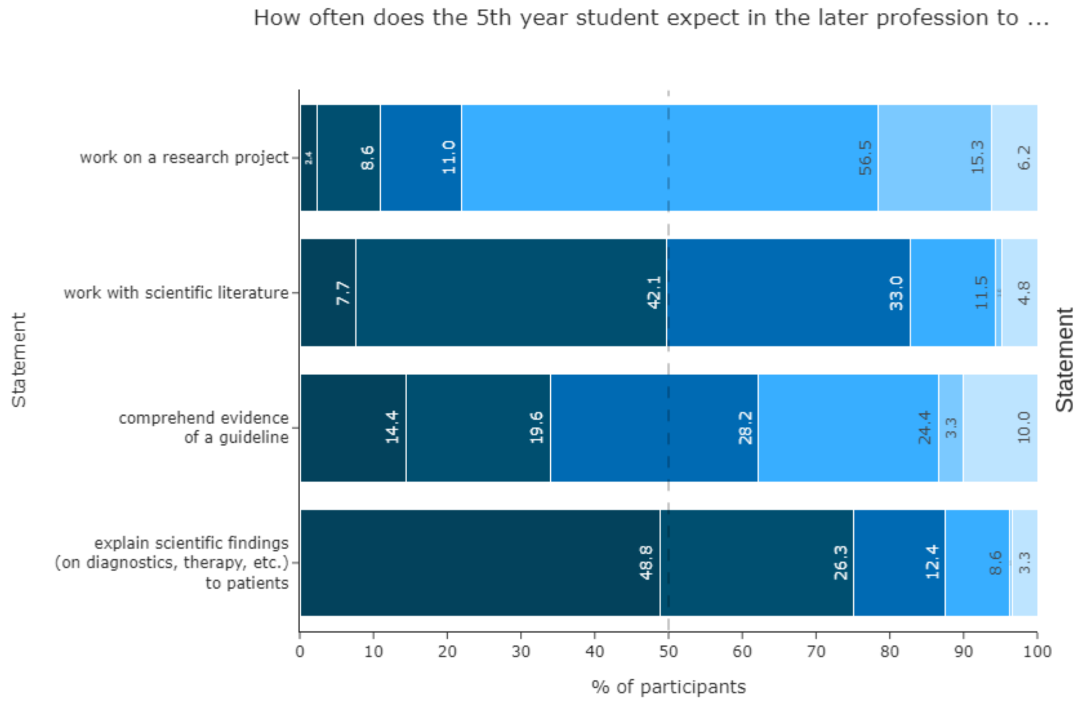


with 856 (only complete answers)

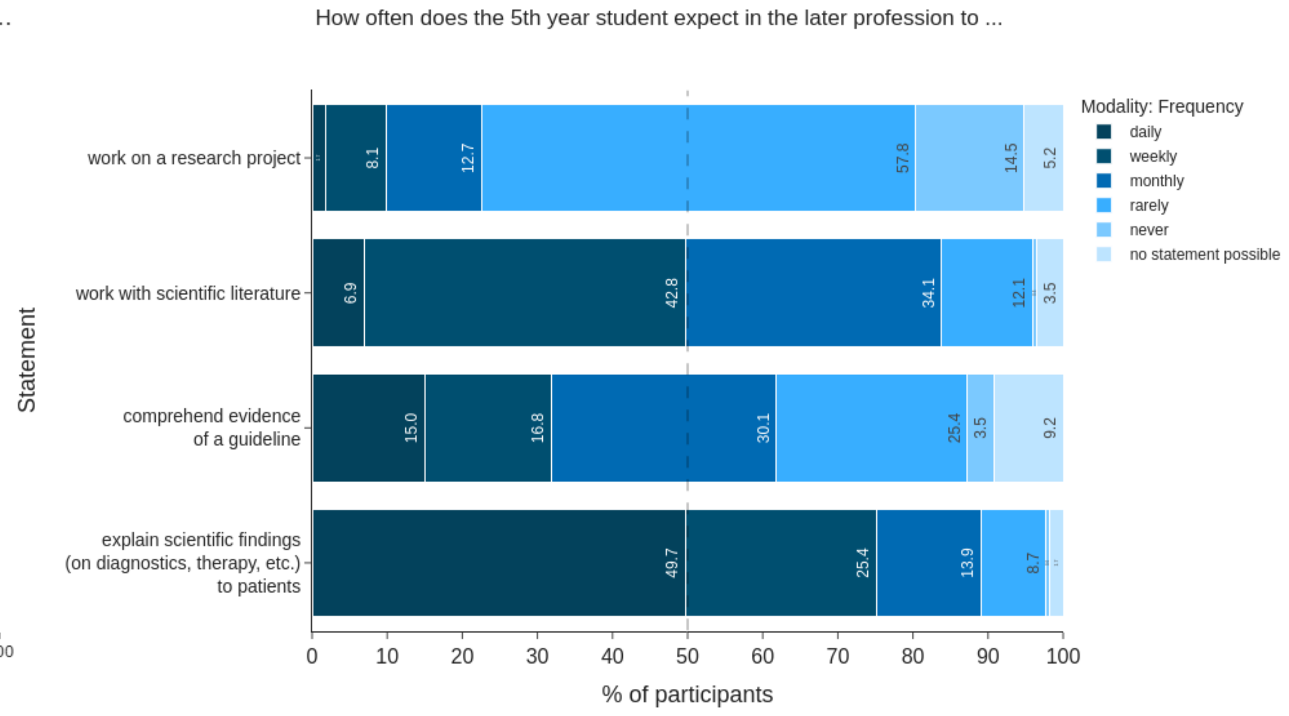

with 980 (all those who have answered this question)

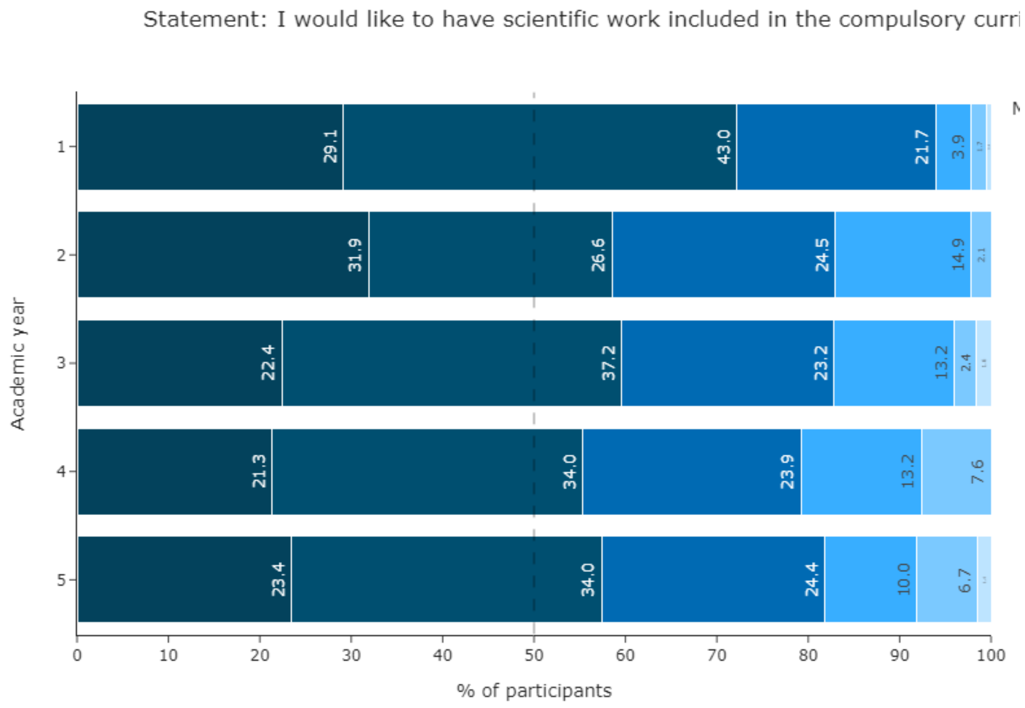


with 856 (only complete answers)

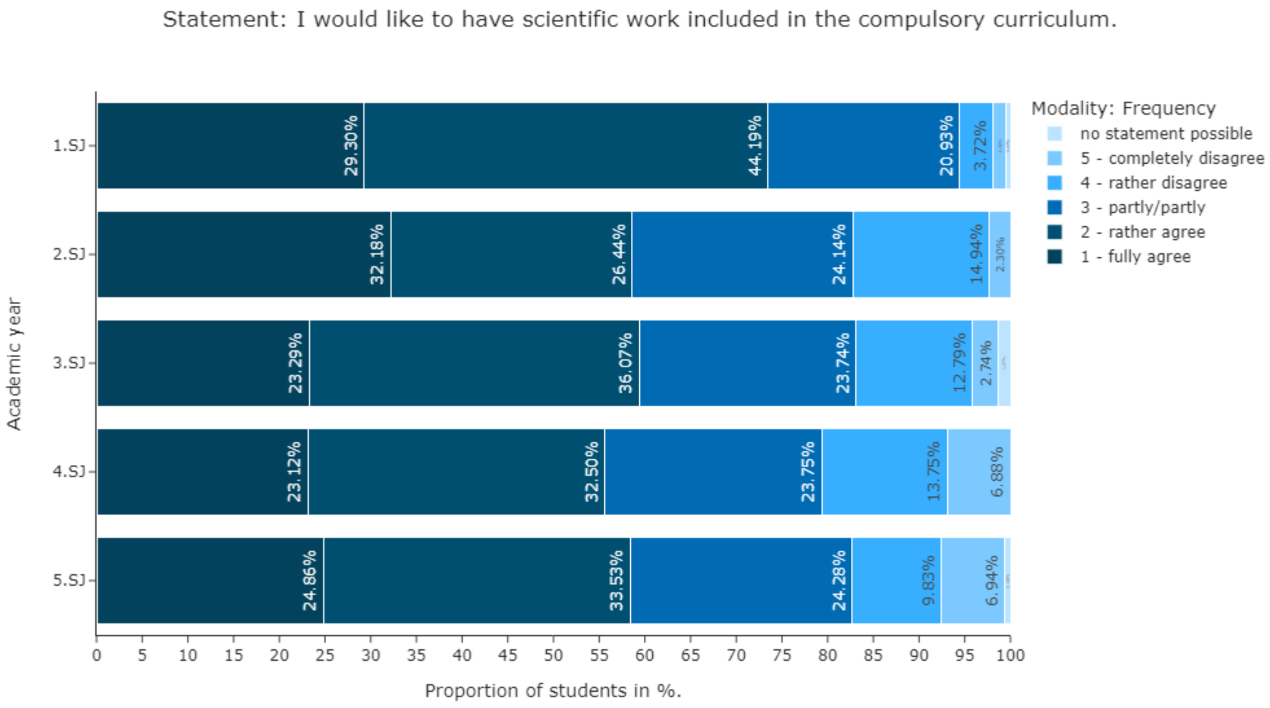

with 980 (all those who have answered this question)

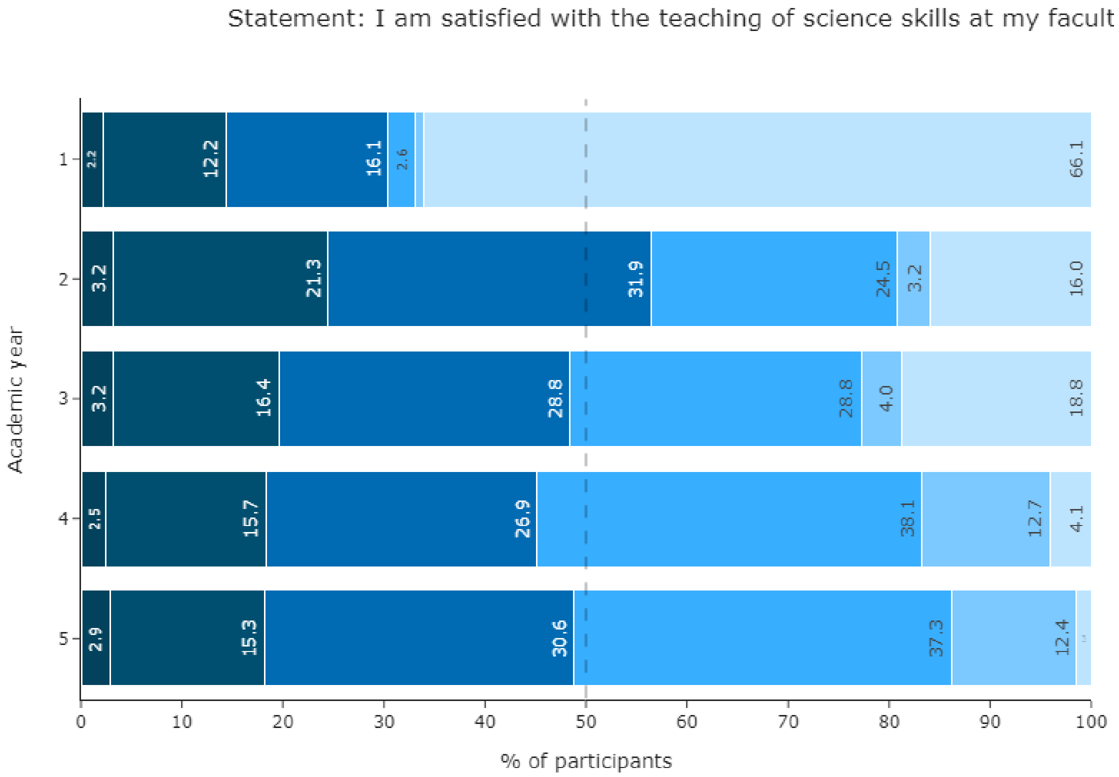


with 856 (only complete answers)

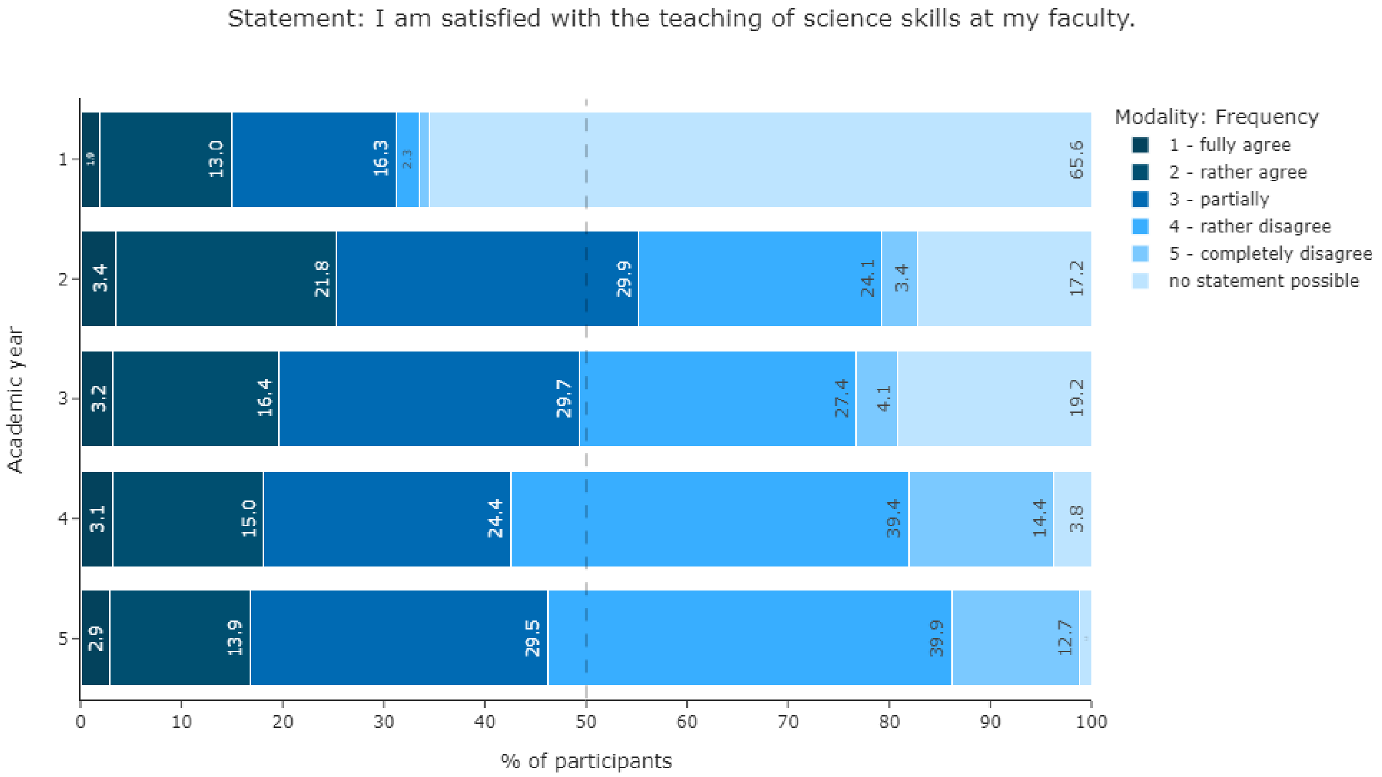

Supplement: Supplementary file 5 — Supplementary Material 5 [file 12909_2024_5470_MOESM5_ESM.pdf]
