# Supplementary material for: Scientific competence during medical education - insights from a cross-sectional study at a German Medical School
Source: BMC Med Educ. 2024 May 28;24:590. doi: 10.1186/s12909-024-05470-7 (PMC11134709; doi:10.1186/s12909-024-05470-7)
Supplement: Supplementary file 6 — Supplementary Material 6 [file 12909_2024_5470_MOESM6_ESM.docx]

Figure Outline

Scientific competence during medical education - insights from a cross-sectional study at a German Medical School

**Table 1 | Survey sample distribution and demographic data per academic year**

|  | 1st academic year | 2nd academic year | 3rd academic year | 4th academic year | 5th academic year |
| --- | --- | --- | --- | --- | --- |
| Enrolled Students | 229 | 223 | 293 | 294 | 294 |
| Participating Students, n | 215 | 87 | 219 | 160 | 175 |
| Response Rate (%) | 93,9 | 39 | 74,7 | 54,4 | 59,5 |
| Sex (n, %) |  |  |  |  |  |
| Male | 65 (30.2%) | 17 (19.5%) | 73 (33.3%) | 48 (30%) | 58 (33.1%) |
| Female | 149 (68.3%) | 70 (80.5%) | 145 (66.2%) | 111 (69.4%) | 116 (66.3%) |
| Diverse | 1 (0.5%) | 0 | 1 (0.5%) | 1 (0.6%) | 1 (0.6%) |
| Age, in years (Mean, SD) | 21.8 ± 2.7 | 23.2 ± 3.7 | 23.1 ± 2.7 | 24.3 ± 3.2 | 25.2 ± 2.9 |
| High School GPA (Mean, SD) | 1.56 ± 0.52 | 1.56 ± 0.48 | 1.54 ± 0.42 | 1.50 ± 0.48 | 1.51 ± 0.40 |
| Promotion (n, %) |  |  |  |  |  |
| Strives for it | 140 (65.1%) | 67 (77.0%) | 173 (79.0%) | 57 (35.6%) | 31 (17.7%) |
| Not planned | 3 (1.4%) | 2 (2.3%) | 5 (2.3%) | 9 (5.6%) | 10 (5.7%) |
| Started | 1 (0.5%) | 1 (1.1%) | 6 (2.7%) | 70 (43.8%) | 113 (64.6%) |
| Don't know | 69 (32.1%) | 17 (19.5%) | 35 (16.0%) | 20 (12.5%) | 18 (10.3%) |
| Cancelled | 0 | 0 | 0 | 2 (1.3%) | 1 (0.6%) |
| Others | 2 (0.9%) | 0 | 0 | 1 (0.6%) | 2 (1.1%) |
| Educational Background (n,%) |  |  |  |  |  |
| None | 81 (37.7%) | 25 (28.7%) | 122 (55.7%) | 96 (60.6%) | 116 (66.3%) |
| Healthcare-related Education | 118 (54.9%) | 59 (67.8%) | 81 (37.0%) | 45 (28.1%) | 42 (24.0%) |
| Healthcare-related Study | 11 (5.1%) | 3 (3.5%) | 10 (4.6%) | 15 (9.4%) | 13 (7.4%) |
| Non-healthcare Education  and Study | 5 (2.3%) | 0 | 6 (2.7%) | 4 (2.5%) | 4 (2.3%) |


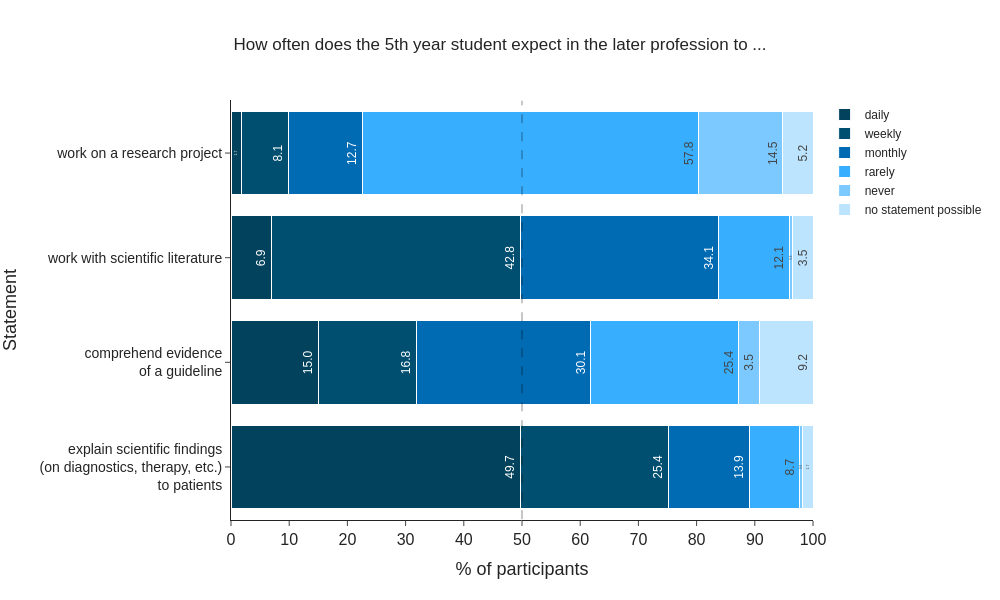


**Figure 1 | Assessment of the need for scientific skills** **among 5th AY students**

Students (n = 175) were asked about specific skills regarding their expected frequency in later professional life. The responses are presented as a stacked bar chart in percentages. The 50% mark is denoted by a dashed line.


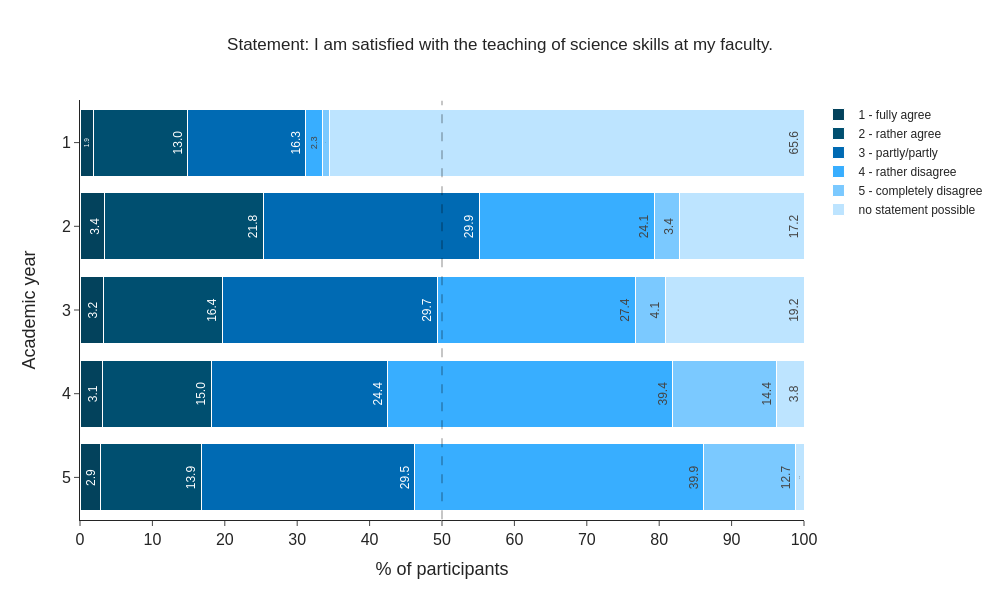


**Figure 2** | **Assessment of satisfaction with the current scientific curriculum across AYs.** The responses of all the students are shown (n = 856). The responses are presented as a stacked bar chart in percentages. The 50% mark is denoted by a dashed line.

**
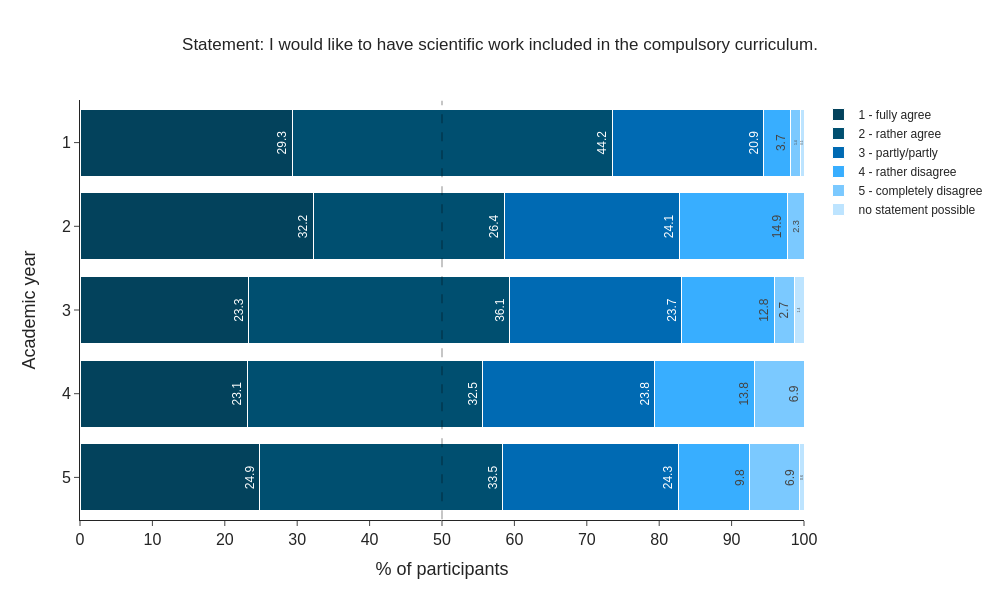
Figure 3 | Evaluation of the introduction of a compulsory curriculum for scientific education.** The answers of all students are shown (n = 856). The answers are shown as a stacked bar chart in percentages. The 50% mark is denoted by a dashed line.

**
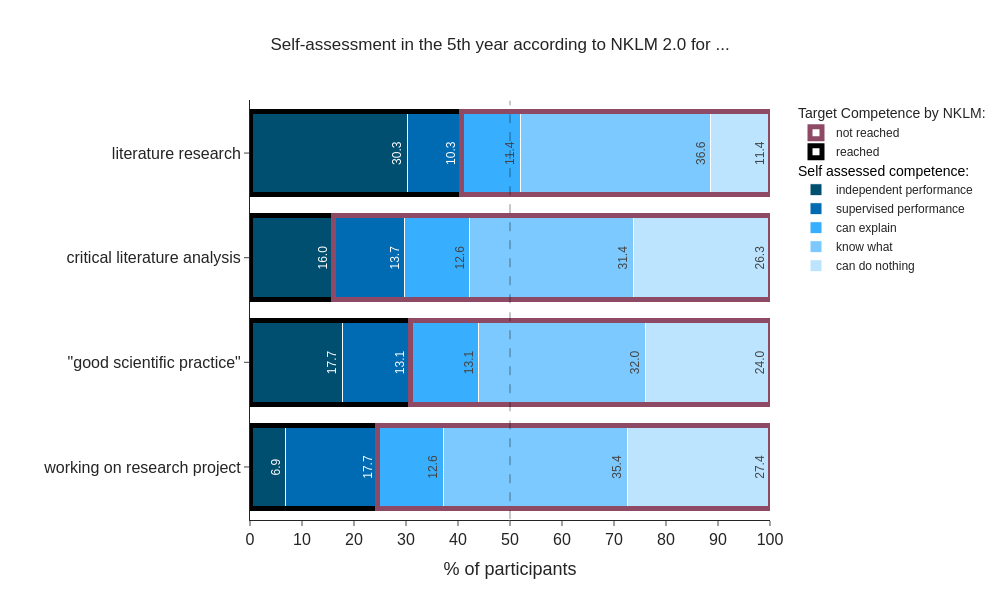
Figure 4 | Self-assessed competencies for different scientific skills in the 5th AY.** The self-assessment is plotted horizontally. The percentage of students who achieved the competence target required is shown by a black outline versus not in red.

**
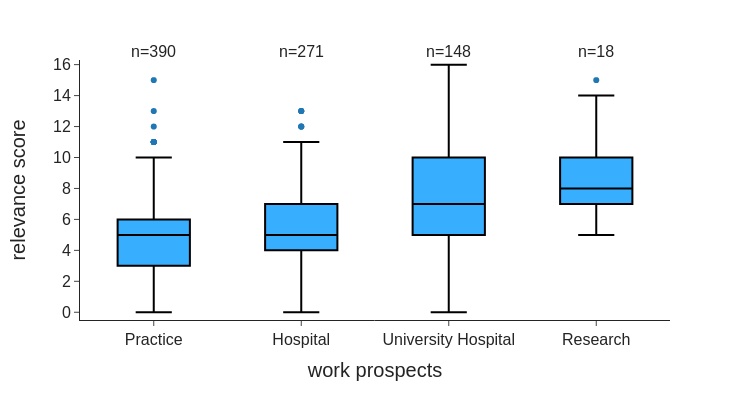
Figure 5 I Box plots for the relevance score of scientific competences (0-16) on later career aspirations.** Assessments of the relevance of scientific competences for later careers: practice (5.0 ± 2.5, n = 390), clinic (5.4 ± 2.5, n = 271), university hospital (7.6 ± 3.4, n = 148), and research (8.7 ± 2.7, n = 17). Significant differences among these groups were found, with specific differences identified between certain categories.

**
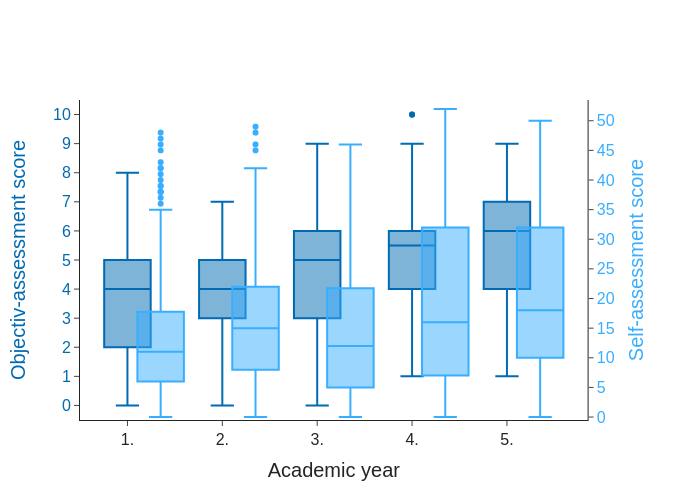
Figure 6 I Box plots for objective and self-assessment score distributions**

The box plots show both the objective assessment (0-10) in dark blue on the left y-axis and the subjective assessment (0-52) in light blue on the right y-axis. The distribution of scores is plotted over the academic years.

**
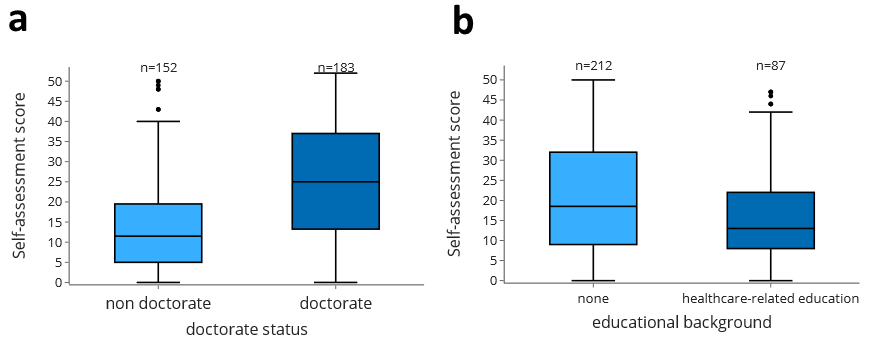
Figure 7 I Factors that influence the self-assessment score**

A) Box plots for self-assessed competences (0-52 points) by doctoral status in the 4th and 5th AY (Non-doctoral students vs. current doctoral students) B) Box plots for self-assessment of competences by educational background in the 4th and 5th AY of study. Students with no previous education and healthcare-related education were compared.

**
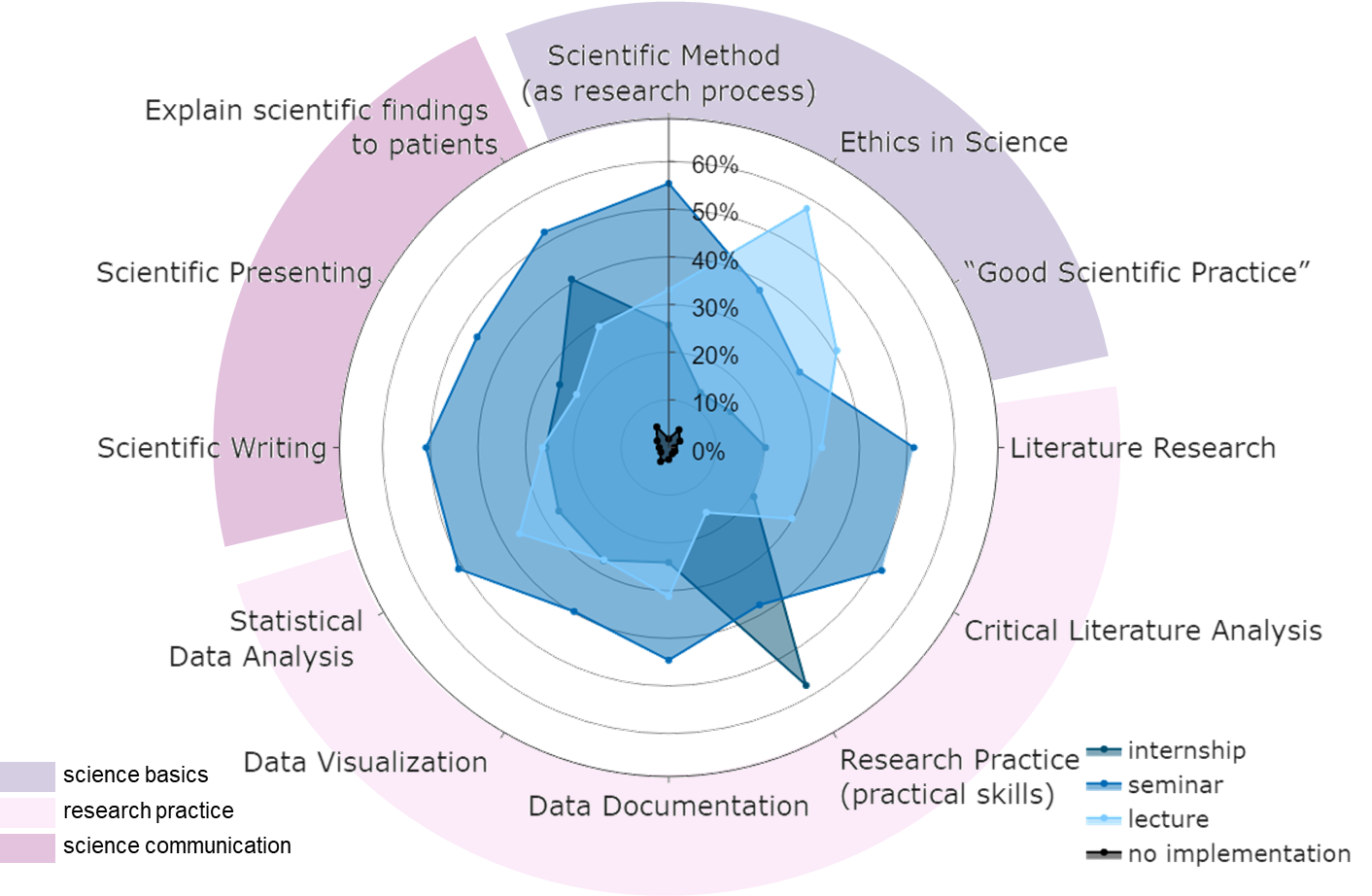
Figure 8 I Students’ wishes for a scientific curriculum**The polar diagram shows the rankings of 12 items for a planned science curriculum as rated by students (multiple choices possible) for implementation in the curriculum (bottom right legend). The items were grouped into 3 content blocks (bottom left legend).
