# Supplementary material for: Scientific competence during medical education - insights from a cross-sectional study at a German Medical School
Source: BMC Med Educ. 2024 May 28;24:590. doi: 10.1186/s12909-024-05470-7 (PMC11134709; doi:10.1186/s12909-024-05470-7)
Supplement: Supplementary file 1 — Supplementary Material 1 [file 12909_2024_5470_MOESM1_ESM.pdf]

Survey of scientific competence among medical students in Dresden, winter semester of 2022.

Survey of scientific competence among students at Dresden Medical School

Dear students,

Thank you for taking the time to complete our survey on scientific work.

During the course of our studies and professional life, we all come into contact with scientific literature or will work scientifically - whether through a doctoral degree, a guideline or simply out of interest. Scientific competence is therefore an important educational goal in medical school!

With our survey, we would like to ascertain your assessments and existing knowledge on the topic of scientific competence and ask you what you would like or would have wished for in terms of teaching the basics of scientific work during your studies. We are surveying all medical students at the Dresden site and will repeat this over several years to find out how your assessment and knowledge change over the years.

The survey will take about 15 minutes of you time. Your answers will be kept anonymous and strictly confidential. The goal is to use the results to further build a longitudinal science literacy curriculum according to your needs and to prepare you in the best possible way for your later professional life and, if applicable, for your doctoral degree. Thank you very much for your support!

Nadja Jahn (9. semester), Maximilian Vogt (7. semester), Enrik Geißler (7. semester), Jean Paul Bereuter (7. semester), PD Dr. Ingmar Glauche (IMB), Dr. Lydia Günther (medical biology, Project Coordinator Longitudinal Curriculum Science Competence LCWiss)

There are 29 questions in this survey.

personal data

How old are you? *

Your answer must be between 0 and 120
Only an integer value may be entered in this field.
Please write your answer here:

Which gender would you assign yourself to? *

Please choose **only one** of the following:

- male
- female
- diverse
- no specification

What grade did you graduate from high school with? *

Please choose **only one** of the following:

- 0,9
- 1,0
- 1,1
- 1,2
- 1,3
- 1,4
- 1,5
- 1,6
- 1,7
- 1,8
- 1,9
- 2,0
- 2,1
- 2,2
- 2,3
- 2,4
- 2,5
- 2,6
- 2,7
- 2,8
- 2,9
- 3,0
- 3,1
- 3,2
- 3,3
- 3,4
- 3,5
- 3,6
- 3,7
- 3,8
- 3,9
- 4,0
- no specification
- Other

Have you already started or completed an apprenticeship/study before medical school? *

Please choose **only one** of the following:

- yes
- no

All apprenticeships/studies that were pursued for more than 1 semester count.

What study/apprenticeship have you completed? *

Only answer this question if the following conditions are met:
Answer was 'yes' at question '4 [P020]' (Have you already started or completed an apprenticeship/study before medical school?)

Please write your answer here:

How long have you been doing your study/apprenticeship? *

Only answer this question if the following conditions are met:
Answer was 'yes' at question '4 [P020]' (Have you already started or completed an apprenticeship/study before medical school?)

Only an integer value may be entered in this field.
Please write your answer here:

data in years

What semester are you currently in? *

Please choose **only one** of the following:

- 1. semester
- 2. semester
- 3. semester
- 4. semester
- 5. semester
- 6. semester
- 7. semester
- 8. semester
- 9. semester
- 10. semester
- 11. semester
- 12. semester
- 13. semester
- >13. semester
- not associated with any semester

Neither previous courses of study nor semesters on leave/research semesters count. If you are currently on leave of absence, tick the last completed semester.

With what grade did you pass your preliminary medical examination (Physikum)?

Only answer this question if the following conditions are met:
Answer was '13. semester ' *or* '5. semester' *or* '9. semester' *or* '8. semester' *or* '7. semester' *or* '>13. semester ' *or* '6. semester' *or* '10. semester' *or* '11. semester' *or* '12. semester' *or* 'not associated with any semester' at question '7 [P010]' (What semester are you currently in?)

Please choose **only one** of the following:

- 1,0
- 1,5
- 2,0
- 2,5
- 3,0
- 3,5
- 4,0
- no specification
- Other

Which field would you most like to work in later?
*

Please choose **only one** of the following:

- Establishment
- Clinic
- University clinic
- Public Health Service
- Free economy
- Politics
- Research (institutes such as Max-Planck-Institute)
- Other

About the doctoral degree

What stage is your doctoral degree at? *

Choose one of the following answers
Please choose **only one** of the following:

- I am aiming for it.
- I have started it.
- I have completed it.
- I have cancelled it.
- I am still undecided.
- I do not want to pursue a doctoral degree.
- Other

In which field are you doing/have you done your doctoral degree?

Listed below are the three doctoral committees to which all medical doctoral degrees are assigned.
*

Only answer this question if the following conditions are met:
Answer was 'I have started it.' *or* 'I have completed it.' *or* 'I have cancelled it. ' at question '10 [Z010]' (What stage is your doctoral degree at?)

Choose one of the following answers
Please choose **only one** of the following:

- Doctoral degree committee for clinical/conservative subjects
- Doctoral degree committee for clinical/surgical subjects
- Doctoral degree committee for experimental/theoretical subjects
- Other

How satisfied are you ... *

Only answer this question if the following conditions are met:
Answer was 'I have started it.' *or* 'I have completed it.' *or* 'I have cancelled it. ' at question '10 [Z010]' (What stage is your doctoral degree at?)

Please choose the appropriate response for each item:

	very satisfied	rather satisfied	partly/partly	rather unsatisfied	very unsatisfied	no specification
with your results						
with your supervision						
with your topic						

How would you rate the impact of completing a medical doctoral degree on the areas listed below?

1 = no impact, 10 = strong impact *

Please choose the appropriate response for each item:

	1	2	3	4	5	6	7	8	9	10
on the quality of patient care										
on the career										
on the salary										
on the social reputation										

1 = no impact, 10 = strong impact

Expectations of studies and career path

In your later professional life, how often do you expect to ...? *

Please choose the appropriate response for each item:

	never	rarely	monthly	weekly	daily	I can not estimate
work with scientific literature						
work on a research project						
apply statistical methods						
write scientific papers						
present posters and talks						
work on a guideline						
understand the evidence of a guideline						
explain scientific findings (on diagnostics, therapy, etc.) to patients.						

How would you rate your current level of knowledge on the following topics?

The competence levels are based on the NKLM 2.0, whereby the competence level 1 corresponds to factual knowledge, 2 to action and reasoning knowledge, 3a to action competence under guidance/control, 3b to action competence without guidance. *

Please choose the appropriate response for each item:

	0 - can not yet do anything	1 - know and name (what)	2 - explain (how and why)	3a - perform under guidance	3b - perform independently
research questions and hypotheses					
literature research					
critical literature analysis					
statistical data analysis					
scientific writing					
scientific presenting					
data documentation					
data visualisation					
rules of "good scientific practice"					
ethics in science					
study design					
scientific work/research project					
career paths in science					

The competence levels are based on the NKLM 2.0, whereby the competence level 1 corresponds to factual knowledge, 2 to action and reasoning knowledge, 3a to action competence under guidance/control, 3b to action competence without guidance.

Which topics for the acquisition of scientific competencies should be anchored in the curriculum and in what form? *

	lectures	seminar	practicals	elective course offer	obligatory for doctoral degrees	facultative	not anchored	no statement possible
scientific method (as a research process)								
research practice (practical skills)								
literature research								
critical literature analysis								
data documentation								
data visualisation								
statistical data analysis								
scientific writing								
scientific presenting								
communication of scientific knowledge to patients								
communication of local doctoral degree regulations								
subject-specific guidelines								
ethics in science								
"good scientific practice"								
study design								
scientific work/research project								
career paths in science								

How would you rate the following statements? *

Please choose the appropriate response for each item:

	1 - fully agree	2 - tend to agree	3 - partly/partly	4 - tend to not agree	5 - do not agree at all	no statement possible
I would like to be taught scientific work in the mandatory curriculum.						
I am satisfied with the teaching of science skills at my faculty.						
I would like to be taught more skills for scientific work in general.						
The COVID-19 pandemic has increased the relevance of scientific competencies for medical students.						

Competence test

In the following, we would like to ask you 10 questions on various topics to find out your current level of knowledge. These cover a wide range of topics from science theory to doctoral studies and the results are helpful for us to build up the Longitudinal Curriculum Scientific Competence (LCWiss).

Abstracts have a great importance for the application for a conference contribution and for publication databases. What is typically not part of an abstract text?
*

Choose one of the following answers
Please choose **only one** of the following:

- introduction/background
- methods
- results
- discussion/conclusion
- conflicts of interest/sponsoring

As part of a study on the efficiency of a neuroleptic drug, you are to implement randomization. How do you proceed when assigning patients?
*

Choose one of the following answers
Please choose **only one** of the following:

- Patients will be randomly selected for the study.
- Patients are randomly assigned to the investigator.
- During recruitment, patients will first be assigned to the placebo group, and subsequent patients will be assigned to the verum group.
- Patients are randomly assigned to the nocebo group or placebo group.
- Patients are randomly assigned to the verum group or the placebo group.

During a consultation, you explain to the patient that the study situation for this therapy corresponds to evidence level Ia (according to the Oxford Centre for Evidence-based Medicine) and what this means. *

Choose one of the following answers
Please choose **only one** of the following:

- A review of documented case series was systematically performed.
- A systematic review of randomized-controlled trials was performed.
- A review of case-control studies was systematically performed.
- A review of randomized expert opinion was systematically performed.
- A review of independent cohort studies was systematically conducted.

The "Exam grade" is an example of a characteristic with the following scale type: *

Choose one of the following answers
Please choose **only one** of the following:

- ordinal scale
- nominal scale
- interval scale
- ratio scale
- zero scale

Which of the examples is correct according to the citation guidelines of the Medical Faculty Dresden (TU Dresden) for doctoral degrees? *

Choose one of the following answers
Please choose **only one** of the following:

- Respiratory drive by alveolar CO2 accumulation (Müller et al.) leads to long-term maintenance of spontaneous breathing in COPD patients (Kaiser).
- Respiratory drive by alveolar CO2 accumulation leads to long-term preservation of spontaneous breathing in COPD patients* (Kaiser, 2014)(Müller et al.,2003).
- Respiratory drive by alveolar CO2 accumulation leads to long-term maintenance of spontaneous breathing in COPD patients* (Kaiser, 2014; Müller et al.,2003).
- Respiratory drive by alveolar CO2 accumulation [1] leads to long-term preservation of spontaneous breathing in COPD patients (Kaiser, 2014).
- Respiratory drive by alveolar CO2 accumulation [1] leads to long-term preservation of spontaneous breathing in COPD patients [2].

Which of the following methods plays the greatest role in mitigating bias in science? *

Choose one of the following answers
Please choose **only one** of the following:

- double blinding
- ombudsperson
- peer review process
- case rate planning
- anonymization

Which statement about securing good scientific practice is most likely to be correct? *

Choose one of the following answers
Please choose **only one** of the following:

- Original data are subject to confidentiality and are the personal property of the scientists.
- Original data must be stored in a secure location for at least 20 years.
- The Dean of Research is, by virtue of his/her office, the ombudsperson for the preservation of good scientific practice.
- All authors agree on the final version of a manuscript to be published.
- Working group leaders have an honorary authorship and are the last authors of a publication.

"Heresies of science take 50 years to be superseded by new findings, because not only the old professors but also their students must die out." This quote from Max Planck is most likely a *

Choose one of the following answers
Please choose **only one** of the following:

- Premise
- Definition
- Thesis
- Hypothesis
- Theory

Which statement about the inductive research approach is most likely to be true?
*

Choose one of the following answers
Please choose **only one** of the following:

- The focus is on a hypothesis to be tested.
- This approach is used in empirical studies with an already existing data set.
- There is already a lot of prior knowledge about the research topic.
- Inductive research is strongly goal-directed and method-guided.
- It is concluded from a model/theory to the individual case.

Which statement about the doctoral degree regulations at the Medical Faculty Dresden is true? *

Choose one of the following answers
Please choose **only one** of the following:

- Doctoral dissertations must be submitted digitally.
- A course on good scientific practice must be taken.
- A course on case rate planning must be taken.
- It must have been published in a peer-reviewed journal before submission.
- The doctoral degree committee is proposed by the student.

Using the LCWiss OPAL course

There is a freely available offer on the basics of scientific work in OPAL at our faculty. This offer is part of the Longitudinal Curriculum Scientific Competence (LCWiss). All students of the Medical Faculty Dresden are automatically enrolled on the OPAL course.

I used the LCWiss-OPAL course last year ... . *

Only answer this question if the following conditions are met:
Answer was '8. semester' *or* '7. semester' *or* '6. semester' *or* '5. semester' *or* '4. semester' *or* '10. semester' *or* '9. semester' *or* '11. semester' *or* '12. semester' *or* '13. semester ' *or* 'not associated with any semester' *or* '>13. semester ' *or* '3. semester' at question '7 [P010]' (What semester are you currently in?)

Choose one of the following answers
Please choose **only one** of the following:

- never
- once
- multiple times
- often/regularly
- don't know at all

I used the OPAL course ... .

Only answer this question if the following conditions are met:
Answer was 'once' *or* 'multiple times' *or* 'often/regularly' *or* 'don't know at all ' at question '28 [LCW1]' (I used the LCWiss-OPAL course last year ... .)

Check all that apply
Please choose **all** that apply:

- as part of an elective course offer
- as part of a mandatory course
- as part of the doctoral degree
- out of interest

Thank you for completing our scientific competence survey.

Please do not share the contents of the survey with any third parties so as not to influence the data collection.

If you have any questions or comments, please write to lcwiss@tu-dresden.de.

We welcome your feedback.

Submit your survey.
Thank you for completing this survey.
